# Synthesis, Electronic, and Antibacterial Properties of 3,7-Di(hetero)aryl-substituted Phenothiazinyl *N*-Propyl Trimethylammonium Salts

**DOI:** 10.3390/molecules29092126

**Published:** 2024-05-03

**Authors:** Hilla Khelwati, Lasse van Geelen, Rainer Kalscheuer, Thomas J. J. Müller

**Affiliations:** 1 Institute of Organic Chemistry and Macromolecular Chemistry, Faculty of Mathematics and Natural Sciences, Heinrich Heine University Düsseldorf, Universitätsstrasse 1, D-40225 Düsseldorf, Germany; hilla.khelwati@hotmail.com; 2 Institute of Pharmaceutical Biology and Biotechnology, Faculty of Mathematics and Natural Sciences, Heinrich Heine University Düsseldorf, Universitätsstrasse 1, D-40225 Düsseldorf, Germany; lasse.geelen@hhu.de (L.v.G.); kalscheu@hhu.de (R.K.)

**Keywords:** antibacterials, biological properties, cyclic voltammetry, fluorescence, heterocycles, Suzuki coupling

## Abstract

In this study, a library of 3,7-di(hetero)aryl-substituted 10-(3-trimethylammoniumpropyl)10*H*-phenothiazine salts is prepared. These title compounds and their precursors are reversible redox systems with tunable potentials. The Hammett correlation gives a very good correlation of the first oxidation potentials with σ_p_ parameters. Furthermore, the title compounds and their precursors are blue to green-blue emissive. Screening of the salts reveals for some derivatives a distinct inhibition of several pathogenic bacterial strains (*Mycobacterium tuberculosis*, *Staphylococcus aureus*, *Escherichia coli*, *Aconetobacter baumannii*, and *Klebsiella pneumoniae*) in the lower micromolar range.

## 1. Introduction

Phenothiazine, i.e., 10*H*-dibenzo[b,e][1,4]thiazine, is a synthetic non-naturally occurring tricyclic heterocycle that has received recognition in many fields of molecular science, starting from dye chemistry in the late 19th century, via histochemical staining (as dye methylene blue), to pharmacology and biomedicine in the 20th century [1]. In the past decades, the extraordinary electronic structure of this electron-rich system has paved new alleys to functional materials in optoelectronics [2,3], dye-sensitized solar cells [4], and mesoporous silica hybrid materials [5]. And again, in the 21st century, the phenothiazine scaffold is receiving considerable attention for its multifaceted biological activity [6]. Phenothiazine derivatives exhibit promising antibacterial, antifungal, anticancer, antiviral, anti-inflammatory, antimalarial, antifilarial, trypanocidal, anticonvulsant, analgesic, immunosuppressive, and multidrug resistance reversal properties [7]. Increasingly, for phenothiazines, known as non-steroidal anti-inflammatory drugs or local anesthetics, antibacterial activity has been recognized [8]. In addition, among other privileged scaffolds, phenothiazines are considered to be photosensitizers in antimicrobial photodynamic therapy [9]. Derivatives bearing halogens or electron-withdrawing groups have been shown to have anticancer and antiprotozoal effects [10]. Diverse biological activities associated with phenothiazine hybrids make them particularly promising candidates for drug development [11].

For combatting multidrug resistance in treating infectious diseases and cancer, phenothiazines are also discussed as a cost-effective strategy against this resistance; however, simultaneously, their potential effects on bacterial resistance and dysbiosis have been considered [12]. In particular, in targeting Methicillin-Resistant *Staphylococcus aureus* (MRSA) with its multidrug resistance mechanisms, including efflux pumps and biofilm formation, phenothiazines, like chlorpromazine, which has received classic status in chemical neuroscience [13], can be considered to be efflux pump inhibitors [14].

In Gram-negative bacteria, e.g., *Escherichia coli* and *Pseudomonas aeruginosa*, the outermost membrane apparently forms a permeability barrier, causing their already intrinsic resistance to many active substances [15]. Molecules with a cationic or cationic–hydrophobic character can be considered for interaction with the phospholipid membrane of a bacterium.

Cationically substituted phenothiazines are (a) water-soluble and (b) amphiphilic due to the organic residue. In addition, the organic residue is (c) a chromophore (with absorption in near UV/Vis and emission in blue-green (fluorescence microscopy)) and (d) a redox system that is also capable of photo-induced electron transfer. Recently, we showed that phenothiazines with quaternary ammonium groups in their side chains are readily immobilized on both periodic mesoporous organosilica (PMO) and neat silica SBA-15 materials via ion exchange [16]. UV irradiation induced the formation of stable phenothiazine radical cations embedded in the hybrid materials, confirmed by cyclic voltammetry of the native materials and EPR spectroscopy of the oxidized specimen. Here, we generalize the modular approach to the synthesis of 3-trimethylammoniumpropyl sidechain-substituted 3,7-di(hetero)aryl-substituted phenothiazine to provide electronically diverse substitution in positions 3 and 7. Furthermore, we present and discuss the electronic properties investigated with cyclic voltammetry, optical absorption, and emission spectroscopy, and we disclose a screening of the antibacterial activity against seven selected pathogenic strains.

## 2. Results and Discussion

### 2.1. Synthesis

**Scheme 1:**
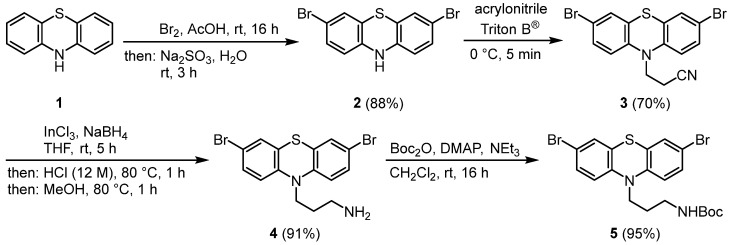
Synthesis of *tert*-butyl (3-(3,7-dibromo-10*H*-phenothiazin-10-yl)propyl)carbamate (**5**).

As in a previous work [16], the synthesis commences with the dibromination of phenothiazine (**1**) to give 3,7-dibromophenothiazine (**2**) in 88% yield (Scheme 1) [17]. The Michael addition of compound **2** in Triton B^®^ furnishes 10-propionitrile phenothiazine **3** in 70% yield [16]. The indium-trichloride-mediated boranate reduction [18] gives 10-(3-amino propyl) 3,7-dibromophenothiazine (**4**) in 91% yield, that is, Boc-protected under standard conditions, to give the dibromo substrate **5** in 95% yield.

**Scheme 2:**
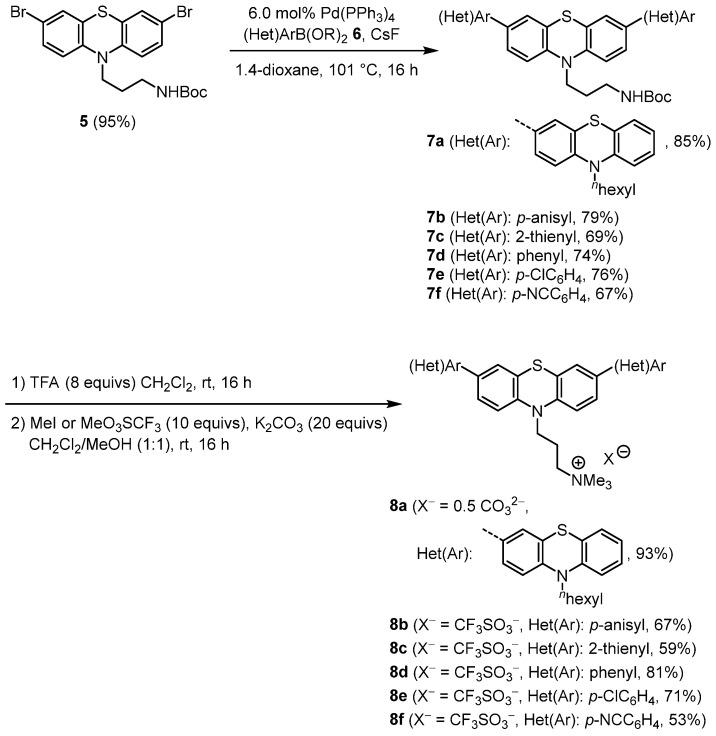
Synthesis of Boc-protected 3,7-di(hetero)aryl-substituted 10-(3-aminopropyl) 10*H*-phenothiazines **7** and 3,7-di(hetero)aryl-substituted 10-(3-trimethylammoniumpropyl)10*H*-phenothiazine salts **8**.

The Boc-protected 10-(3-amino propyl) 3,7-dibromo phenothiazine **5** sets the stage for twofold Suzuki coupling with (hetero)aryl boronic acids or pinacol boronates **6** to give six 3,7-di(hetero)aryl-substituted phenothiazines **7** in good yield after isolation by flash chromatography (Scheme 2). The treatment of compounds **7** with an excess of trifluoroacetic acid leads to Boc deprotection; the ammonium salt is permethylated in the presence of an excess of methyl iodide (for the preparation of salt **8a**) or methyl triflate (for the preparation of salts **8b–f**), and potassium carbonate as a base gives rise to the isolation of six salts **8** in moderate to excellent yield. The structures of the salts (compound **8a** as a carbonate with two cations and compounds **8b–f** as triflates with one cation) is unambiguously assigned by the combination of ^1^H and ^13^C NMR spectroscopy, indicating mirror plane symmetry by the occurrence of a reduced set of signals, with IR spectroscopy identifying the counter anion by characteristic vibration bands (1454 cm^−1^ as a very dominant CO valence vibration for the carbonate ion in salt **8a**; ~1240 and ~1030 cm^−1^ as very dominant SO and CF valence vibrations for triflate ions in the salts **8b–f**), mass spectrometry unambiguously verifying the molecular peak of the cation, as well as HRMS identifying the molecular composition of the cations and/or combustion analysis for the molecular composition of the salts.

### 2.2. Electronic Properties of Compounds ***7*** and ***8***

As part of our ongoing interest in the structure–property relationships of 3-(hetero)aryl and 3,7-di(hetero)aryl-substituted phenothiazines [19,20], we became intrigued in studying the effect of Boc-protected 3-aminopropyl (compounds **7**) and 3-trimethylammoniumpropyl sidechains (compounds **8**) at the 10-position of 3,7-di(hetero)aryl-substituted phenothiazines on the electronic properties in the electronic ground and excited states. Therefore, cyclic voltammograms and absorption and emission spectra were recorded to determine the redox potentials (cyclic voltammograms) and UV/Vis absorption and fluorescence bands and thereof the Stokes shifts (Table 1).

The cyclic voltammetry of triads **7a** and **8a** discloses the expected behavior of three conjugatively coupled electrophores [21,22] with three reversible one-electron oxidations at potentials of 0.620, 0.740, and 0.850 V (**7a**) and 0.610, 0.720, and 0.830 V (**8a**), respectively (Table 1). As can be seen by the minute differences in the oxidation potentials, the effect of the remote 3-NH-Boc or 3-trimethylammonium substituent on the sidechain is only minor. Indeed, it can be considered that the first oxidation giving a radical cation, that is delocalized over all three phenothiazines, renders this potential to lie cathodically shifted. For the consanguineous series **7b–f** and **8b–f**, the site of oxidation is clearly localized on the central phenothiazine unit, which certainly experiences the Coulombic interaction of the sidechain after oxidation. The first oxidation potentials *E*_0_^0/+1^ are found in a range from 0.640 to 0.830 V (compounds **7b–f**) and from 0.680 to 0.840 V (compounds **8b–f**), respectively. The second oxidations to the phenothiazine-centered dications are only found at potentials *E*_0_^+1/+2^ between 1.040 and 1.400 V for the more electron-rich substituted compounds **7b–d** and **8b–d**. For both series, a distinct anodic shift of *E*_0_^0/+1^ is discernable with altering the remote 3,7-substituents from electron-releasing to electron-withdrawing. For a closer inspection to estimate the transmission of the polar substituent effect of the remote substituent in the 3- and 7-position on the ease of oxidation, correlation analyses of the oxidation potentials *E*_0_^0/+1^ with Hammett parameters [23] σ_p_, σ_R_, σ_R+_, σ_p+_, and σ_p−_ were considered.

In the consanguineous series of compounds **7b–f**, indeed, the highest linear regression coefficients are obtained for parameters σ_p_ (R^2^ = 0.9903), σ_p+_ (R^2^ = 0.9631), and σ_p−_ (R^2^ = 0.9491) (for details, see Supporting Information). The involvement of σ_p_ clearly indicates that the electronic substituent effects are transmitted via resonance and inductive pathways, as previously observed for other 3,7-disubstituted phenothiazines [19,24]. The same correlation can be established for the consanguineous series of the trimethylammonium salts **8b–f**, where the linear regression coefficients for σ_p_ (R^2^ = 0.9855) and σ_p+_ (R^2^ = 0.9839) are very similar and for σ_p−_ (R^2^ = 0.9056) are less, indicating the similar transmission mechanism of remote polar substituents by resonance and inductive pathways for the stabilization of the formed radical dication.

**Figure 1:**
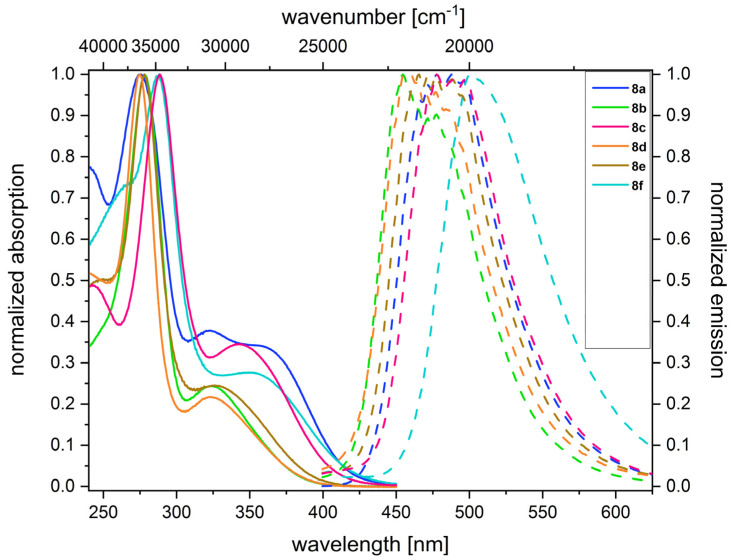
Normalized absorption (solid) and emission (dashed) spectra of 3,7-di(hetero)aryl-substituted 10-(3-trimethylammoniumpropyl)10*H*-phenothiazine salts **8** (recorded at *T* = 298 K for *c*(**8**) = 10^−3^ m (absorption) and *c*(**8**) = 10^−6^ m (emission) in dichloromethane).

The absorption spectra disclose the longest wavelength bands between 330 and 373 nm (compounds **7a–f**) and between 323 and 368 nm (compounds **8a–f**), with absorption coefficients in a range between 6000 and 41,000 m^−1^cm^−1^ (Table 1, Figure 1). 

While the red shifts of the triads **7a** and **8a** amount to intense shoulders at 367 nm (**7a**) or a maximum at 368 nm (**8a**) attributed to the extended π-conjugation, the most redshifted bands appear for the *p*-cyano phenyl derivatives at 373 nm (**7f**) and 359 nm (**8f**). All compounds **7** and **8** luminesce intensively with a blue to green color in a range from 455 to 520 nm, and the Stokes shifts are quite broad, ranging from 6300 to 9500 cm^−1^, which is quite typical for phenothiazine as a consequence of geometrical and electronic changes upon photonic excitation, namely the planarization of the phenothiazine core in the vibrationally relaxed excited state and considerable charge transfer from the central phenothiazine donor to the 3,7-substituents that act as acceptors in the excited state [25,26].

Correlation analyses with the photophysical data λ_max,abs_ (absorption), λ_max,em_ (emission), and Δ$\tilde{\nu }$ (Stokes shift) of the consanguineous series of compounds **7b–f** and **8b–f**, with Hammett parameters of σ_p_, σ_R_, σ_R+_, σ_p+_, and σ_p−_, can be likewise probed for estimating the transmission of the polar substituent effect of the remote substituent in 3- and 7-position on excitation, radiative deexcitation, and structural changes in the vibrationally relaxed excited state [20]. While the data of the longest wavelength absorption bands λ_max,abs_ only give mediocre (compounds **7b–f**) or poor (compounds **8b–f**) linear correlations with σ_p−_ and σ_p_, respectively, the emission bands λ_max,em_ correlate for both series with σ_p_ and σ_p-_ reasonably well (compounds **7b–f**, σ_p−_: R^2^ = 0.9564; compounds **8b–f**, σ_p_: R^2^ = 0.9129). This accounts for the transmission of the remote substituent effects in the planarized vibrationally relaxed excited state of phenothiazines [25,27]. Expectedly, the Stokes shifts indicating electronic or geometrical structural changes upon photonic excitation do not correlate in a meaningful sense due to the poor correlation of the absorption bands.

### 2.3. Biological Properties of Compounds ***7*** and ***8***

We have tested the activity of the selected 3,7-di(hetero)aryl-substituted 10-(3-trimethylammoniumpropyl)10*H*-phenothiazine salts **8** and salt **9** [16] in a screening assay against common human pathogens, namely *Mycobacterium tuberculosis*, the Gram-positive bacterium *Staphylococcus aureus*, and four Gram-negative bacteria (*P. aeruginosa*, *E. coli*, *Actinobacter baumannii*, *Klebsiella pneumoniae*), to assess the antibacterial activity, i.e., determine the minimal inhibitory concentration (MIC) against the corresponding bacterial strains (Table 2).

The activity screening reveals that 3,7-diaryl-substituted 10-(3-trimethylammoniumpropyl)10*H*-phenothiazine salts **8b–f** can effectively inhibit the growth of *M. tuberculosis* (MIC 12.5–25 μm) and *S. aureus* (MIC 6.25–12.5 μm), where compound **8d** is the most efficacious. In addition, this compound also inhibits the growth of *E. coli* (MIC 25 μm), *A. baumannii* (MIC 25 μm), and *K. pneumoniae* (MIC 12.5 μm). Apparently, *P. aeruginosa* is not inhibited by any of the substances under a threshold MIC of 100 μm. For *A. baumannii*, the aryl substituents obviously should be small, since bis(phenyl) derivative **8d** (MIC 25 μm) and bis(*p*-chlorophenyl) derivative **8e** (MIC 50 μm) at least show some inhibition. The steric demand predominantly applies for compound **8a**, which, despite its lipophilicity by the two *n*-hexyl phenothiazinyl substituents, does not show any activity of the tested pathogen strains. However, since the smallest compound in this series, compound **9**, also fails almost completely, a balanced decoration rendering lipophilicity and amphiphilicity as for 3,7-diaryl-substituted derivatives proves to give the most efficacious inhibition. This promises a good starting point for suitable cationically substituted phenothiazine derivatives.

## 3. Materials and Methods

### 3.1. General Considerations

Unless otherwise stated, all reactions were carried out in a nitrogen atmosphere using Schlenk, septum, and cannula techniques in heated single- or multi-neck flasks or Schlenk tubes. Nitrogen and argon were used as protective gases. Heated silicone baths were used at higher temperatures. At lower temperatures, cooling baths were used (acetone/dry ice, ice/water, or ice/salt mixtures). A high-vacuum pump from Büchi (Essen, Germany) was used for securation. Solvents were removed by distillation using vacuum pumps and rotary evaporators from Heidolph Instruments GmbH & Co. KG (Schwabach, Germany). The rotary evaporators were operated at a water bath temperature of 40 °C.

The chemicals used for this work, which were not synthesized in-house, were obtained commercially from the companies ABCR GmbH & Co. KG (Karlsruhe, Germany), Acros Organics (Geel, Belgium), Alfa-Aesar GmbH & Co. KG (Ward Hill, MA, USA), Carl Roth GmbH & Co. KG (Karlsruhe, Germany), Macherey-Nagel (Düren, Germany), Merck KGaA (Darmstadt, Germany), Sigma-Aldrich Chemie GmbH (St. Louis, MO, USA), or VWR (Radnor, PA, USA). Boronic acids and boronates **6** were synthesized according to the procedures outlined in the literature. The solvents used were purchased in pure form and removed as far as possible from water and other impurities according to standardized regulations or taken from a solvent drying system MB-SPS-800 from M. Braun Inertgas-Systeme GmbH (Garching, Germany).

For column chromatography, silica gel 60 m from Macherey-Nagel (grain size 0.04–0.063) or from Sigma-Aldrich (St. Louis, MO, USA, mesh 70–230, grain size 0.04–0.063, or mesh 230–400) was used as the stationary phase, and Celite^®^ 545 was used as the adsorbent, and this was obtained from Carl Roth GmbH. Pure sea sand from AppliChem (Chicago, IL, USA) was also used. Column chromatography was carried out using flash technology at an overpressure of two bars of compressed air. Various solvents and solvent mixtures of distilled *n*-hexane, distilled *n*-hexane/acetone, distilled dichloromethane, and distilled dichloromethane/methanol were used as eluents.

The polarity of the eluent and the qualitative monitoring of the reaction progress were determined and carried out using thin-layer chromatography. Here, the R_f_ value of the product was adjusted so that it was approximately in the range of 0.3. For this purpose, silica-yellow-coated aluminum foil (60, F254) from Merck KGaA with a fluorescent indicator was used. Detection was carried out using UV light with wavelengths of 254 nm and 365 nm.

The ^1^H and ^13^C NMR spectra were recorded using the Avance III 300, DRX 500 and Avance III 600 spectrometer from Bruker (Karlsruhe, Germany). The solvents used were acetone-d_6_ and DMSO-d_6_, whereby all ^1^H and ^13^C spectra were locked to the signals of the non-deuterated solvents (acetone-d_6_: ^1^H NMR: *δ* 2.84, ^13^C NMR: *δ* 206. 3 and *δ* 29.8; DMSO-d_6_: ^1^H NMR: *δ* 2.50, ^13^C NMR: *δ* 39.51). The chemical shifts *δ* are given in ppm, and the coupling constants *J* are given in Hz. The individual signals were identified and assigned with the aid of the chemical shifts, the integrals, the multiplicity, and the coupling constants. When describing the multiplicity of the individual signals, the following common abbreviations were used: s (singlet), d (doublet), t (triplet), q (quartet), dd (doublet of doublet), dt (doublet of triplet), quint. (quintet), and m (multiplet). The carbon nuclei were assigned using ^13^C NMR and 135-Dept spectra, with primary carbon nuclei designated as CH_3_, secondary as CH_2_, tertiary as CH, and quaternary as C_quat_.

The electron ionization mass spectra were recorded using the TSQ 7000 triple quadrupole mass spectrometer from Finnigan MAT (Thermo Fisher Scientific, Waltham, MA, USA). The Ultraflex I spectrometer from Bruker DALTONICS was used to record the MALDI (TOF) spectra. High-resolution ESI measurements were carried out on a UHR-QTOF maXis 4G, also from Bruker Daltonics.

The infrared spectra were recorded on an IR-Affinity-1 using the attenuated total reflectance (ATR) technique from Shimadzu (Kyoto, Japan). The solids obtained were plotted and measured as such. The position of the absorption bands in the spectrum was indicated in wavenumbers $\tilde{\nu }$ [cm^−1^]. The intensities of the IR absorption bands obtained are indicated as s (strong), m (medium), and w (weak).

The elemental analyses were carried out using the Perkin Elmer Series II Analyzer 2400 from PerkinElmer (Waltham, MA, USA) or an Elementar vario MICRO CUBE at the Institute for Pharmaceutical and Medicinal Chemistry at Heinrich Heine University Düsseldorf, and the melting points were determined using the Melting Point B-540 apparatus from Büchi.

The UV/Vis spectra were recorded on a UV/VIS/NIR Lambda 19 spectrometer from Perkin Elmer. All compounds were measured at room temperature using high-purity solvents (HPLC or UVASOL). The extinction coefficients were determined using Lambert–Beer’s law. For this purpose, five absorbance spectra of the respective compound were recorded at different concentrations. By plotting the absorbance against the concentration, the absorbance coefficient could be determined from the slope of the equalization line at the selected wavelength.

The emission spectra presented in this work were recorded on a calibrated Hitachi F-7000 fluorescence spectrometer (Tokyo, Japan). The fluorescence was always excited at the absorption maximum. All samples were measured at room temperature in dichloromethane (purity grade HPLC or UVASOL).

All experimentally recorded cyclic voltammograms within this work were measured in a small-volume glass cell (4 mL) with a three-electrode arrangement. The experiments were performed under argon atmosphere in dry and degassed dichloromethane at room temperature with feed rates of ν = 100, 250, 500, and 1000 mV·s^−1^. Tetra-*n*-butylammonium hexafluorophosphate (0.1 m in dichloromethane) was used as the electrolyte. A 2 mm platinum disk coated with glass was used as the working electrode. The counter electrode was a platinum wire, and the reference electrode consisted of a solid Ag/AgCl electrode filled with a sodium chloride solution (3.5 m). All potentials were referenced to the internal standard of decamethylferrocene/decamethylferrocenium ([DMFc]/[DMFc]^+^, *E*_0_^0/+1^ = −0.095 V). The absolute potential of ([DMFc]/[DMFc]^+^ was determined against that of ferrocene ([Fc]/[Fc]^+^, *E*_0_^0/+1^ = 0.450 V) [28]. The redox-active substance was weighed into the measuring cell together with the conducting salt and degassed for five minutes by introducing argon while stirring. The model 263A galvanostat/potentiostat from EG&G Princeton Applied Research (Champaign, IL, USA) was used. The device was controlled by the PowerSuite Revision 2.12.1 program from Princeton Applied Research PerkinElmer Instruments.

### 3.2. Antibacterial Activity Testing

The cultivation of *M. tuberculosis* strain H37Rv was performed in PETG square bottles (Nalgene; Rochester, New York, NY, USA) in Middlebrock 7H9 liquid growth medium (BD; Franklin Lakes, NJ, USA) supplemented with 10% ADS (0.81% NaCl (*w*/*v*), 5% BSA (*w*/*v*), 2% dextrose (*w*/*v*)), 0.5% glycerol (*v*/*v*), and 0.05% tyloxapol (*v*/*v*). The MIC assay was performed in 96-well round-bottom polystyrene plates (Greiner; Kremsmünster, Austria) with a compound test range from 100 µM to 0.78 µM in a total volume of 100 µL. After static incubation for five days at 37 °C (5% CO_2_, 80% humidity), growth was assessed using the resazurin reduction assay. For this, 10 μL of resazurin solution (100 μg/mL, Sigma Aldrich) was added into each well and incubated overnight. After the fixation of cells for 30 min at room temp following the addition of 10% (*v*/*v*) formalin, growth was quantified by measuring fluorescence using a microplate reader (TECAN; Maennedorf, Switzerland) (excitation: 540 nm, emission: 590 nm). After calculating growth relative to the DMSO solvent control (=100% growth) and uninoculated wells (subtraction of background fluorescence = 0% growth), the MIC value was determined as the actually tested lowest compound concentration, which resulted in 10% residual growth or less. Rifampicin was used as a positive control in all the experiments with *M. tuberculosis*.

The growth of the nosocomial bacteria *Staphylococcus aureus* (ATCC 29213), *Pseudomonas aeruginosa* (ATCC 27853), *E. coli* (ATCC 25922), *Acinetobacter baumannii* (ATCC BAA-747), and *Klebsiella pneumoniae* (ATCC 13883) was performed at 37 °C in Mueller–Hinton medium (BD; Franklin Lakes, NJ, USA). Moxifloxacin was used as the positive control. The antimicrobial activity against nosocomial bacteria was determined following the CLSI guidelines [29] in 96-well round-bottom polystyrene plates (Greiner; Kremsmünster, Austria) with a compound test ranging from 100 µm to 0.78 µm in a total volume of 100 µL. The MIC values were determined after overnight incubation as the actually tested lowest compound concentration that prevented visible growth by macroscopical evaluation.

### 3.3. Synthesis of tert-Butyl (3-(3,7-Dibromo-10H-phenothiazin-10-yl)propyl)carbamate *(**5**)* [16]

#### 3.3.1. 3,7-Dibromo-10*H*-phenothiazine (**2**)

Phenothiazine (**1**) (25.0 g, 125 mmol) was placed in a dry two-necked round-bottom flask with a magnetic stir bar under nitrogen, and degassed acetic acid (600 mL) was added. Bromine (13.5 mL, 263 mmol) in acetic acid (200 mL) was added slowly and dropwise from the dropping funnel to the reaction mixture. The deep-colored solution was then stirred at room temperature for 16 h. A saturated aqueous solution of sodium sulfite (31.5 g, 250 mmol) was then added to the reaction mixture, which was then stirred at room temperature for 3 h. The initially violet suspension brightened to milky beige within this time. Then, the reaction mixture was poured on ice water (2 L). The resulting greenish precipitate was collected by suction and washed with a small amount of ice water. The precipitate was dried to weight constancy to give 3,7-dibromo-10*H*-phenothiazine (**2**) (50.7 g, 88%) as a greenish powder, Mp 186–188 °C. *R_f_* (*n*-hexane/acetone 7:3): 0.42.

^1^H NMR (300 MHz, DMSO-d_6_): *δ* 6.58 (d, ^3^*J* = 8.3 Hz, 2 H), 7.11 (d, ^4^*J* = 2.3 Hz, 2 H), 7.14 (dd, ^4^*J* = 2.3 Hz, ^3^*J* = 8.3 Hz, 2 H), 8.84 (s, 1 H). ^13^C NMR (151 MHz, DMSO-d_6_): *δ* 112.7 (C_quat_), 116.0 (C_quat_), 118.2 (CH), 128.1 (CH), 130.3 (CH), 140.9 (C_quat_). MS (EI) *m*/*z* (%): 359 (51, [C_12_H_7_^81^Br_2_NS]^+^), 357 (100, [C_12_H_7_^79^Br^81^BrNS]^+^), 355 (54, [C_12_H_7_^79^Br_2_NS]^+^), 278 (78, [C_12_H_7_^81^BrNS]^+^), 276 (73, [C_12_H_7_^79^BrNS]^+^), 197 (81, [C_12_H_7_NS]^+^). IR $\tilde{\nu }$ [cm^−1^]: 3377 (vw), 3321 (w), 3069 (vw), 2849 (vw), 1566 (vw), 1441 (m), 1420 (m), 1383 (m), 1368 (w), 1288 (m), 1236 (w), 1136 (w), 1080 (m), 1057 (w), 1020 (w), 934 (w), 881 (m), 864 (w), 847 (w), 808 (s), 750 (m), 731 (m), 700 (w), 673 (m), 648 (w).

#### 3.3.2. 3-(3,7-Dibromo-10*H*-phenothiazin-10-yl)propiononitrile (**3**)

3,7-Dibromo-10*H*-phenothiazine (**2**) (21.0 g, 59.0 mmol) was placed in a dry Schlenk flask with a magnetic stir bar under nitrogen and suspended in acrylonitrile (120 mL). The mixture was cooled to 0 °C (ice–water bath), and 40 wt.% of Triton B^®^ in methanol (2.15 mL) was added slowly and dropwise under external cooling and vigorous stirring. After the addition was complete, the reaction mixture was stirred for 5 min. The progress of the reaction was monitored by thin-layer chromatography, and then the reaction mixture was poured into water (480 mL), and a beige solid precipitated. The precipitate was filtered off and dried under vacuum for 16 h. The crude product was adsorbed on Celite^®^, purified by column chromatography on silica gel (*n*-hexane/acetone 8:2), suspended in a small volume of acetone, filtered, and dried under vacuum to weight constancy to give 3-(3,7-dibromo-10*H*-phenothiazin-10-yl)propiononitrile (**3**) (16.9 g, 70%) as a colorless solid, Mp 146–148 °C. *R_f_* (*n*-hexane/acetone 8:2): 0.26.

^1^H NMR (600 MHz, DMSO-d_6_): *δ* 2.90 (t, ^3^*J* = 6.6 Hz, 2 H), 4.18 (t, ^3^*J* = 6.6 Hz, 2 H), 7.04 (d, ^3^*J* = 8.7 Hz, 2 H), 7.39 (dd, ^3^*J* = 8.7, ^4^*J* = 2.3 Hz, 2 H), 7.42 (d, ^4^*J* = 2.3 Hz, 2 H). ^13^C NMR (125 MHz, DMSO-d_6_): *δ* 15.7 (CH_2_), 42.5 (CH_2_), 114.6 (C_quat_), 117.7 (CH), 118.4 (C_quat_), 126.1 (C_quat_), 129.2 (CH), 130.3 (CH), 142.9 (C_quat_). MS (EI) *m*/*z* (%): 412 (37, [M^81^Br_2_]^+^), 410 (81, [M^81^Br^79^Br]^+^), 408 (37, [M^79^Br]^+^), 372 (52, [C_13_H_8_^81^Br_2_N^32^S]^+^), 370 (100, [C_13_H_8_^81^Br^79^BrN^32^S]^+^), 368 (48, [C_13_H_8_^79^Br_2_N^32^S]^+^), 358 (44, [(C_12_H_6_^81^Br_2_N^32^S]^+^), 356 (89, [(C_12_H_6_^81^Br^79^BrN^32^S]^+^), 354 (42, [(C_12_H_6_^79^Br_2_N^32^S]^+^), 291 (59, [C_13_H_8_^81^BrN^32^S]^+^), 289 (54, [C_13_H_8_^79^BrN^32^S]^+^), 196 (65, [C_12_H_6_N^32^S]^+^). IR $\tilde{\nu }$ [cm^−1^] = 3086 (vw), 2990 (vw), 2961 (vw), 2905 (vw), 2249 (vw), 1585 (vw), 1477 (w), 1452 (s), 1408 (w), 1391 (w), 1335 (m), 1317 (m), 1288 (w), 1248 (m), 1227 (w), 1194 (m), 1165 (w), 1155 (w), 1113 (w), 1096 (w), 1080 (w), 1070 (w), 1045 (w), 1028 (vw) 989 (vw), 901 (vw), 864 (m), 797 (s), 789 (m), 752 (m), 741 (w), 700 (vw), 650 (w), 606 (w).

#### 3.3.3. 3-(3,7-Dibromo-10*H*-phenothiazin-10-yl)propylamine (**4**)

Indium trichloride (3.32 g, 15.0 mmol) and sodium borohydride (1.70 g, 45.0 mmol) were suspended in THF (50.0 mL) in a dry Schlenk tube with a magnetic stir bar and stirred at room temp for 1 h. After a careful and portion-wise addition of 3-(3,7-dibromo-10*H*-phenothiazin-10-yl)propylnitrile (**3**) (6.11 g, 15.0 mmol) to the grayish reaction mixture, the reaction mixture was stirred at room temp for 4 h. The reaction was stopped by the careful and dropwise addition of 3.00 m hydrochloric acid (50 mL), resulting in strong gas evolution. The reaction solution was then heated at 90 °C to reflux for 1 h. After cooling to room temp and the addition of methanol (25 mL) to the reaction mixture, heating to reflux at 90 °C was continued for 1 h. After cooling, the volatile organic components were removed under reduced pressure, and the precipitate was separated from the remaining aqueous phase by filtration. The crude product was adsorbed on Celite^®^ and purified by column chromatography on silica gel (*n*-hexane/acetone/triethylamine 7:2:0.1). Then, the product was suspended in a small volume of methanol, filtered, and dried to weight constancy under vacuum to give 3-(3,7-dibromo-10*H*-phenothiazin-10-yl)propylamine (**4**) (6.09 g, 91%) as a pale beige powder, Mp 139–141 °C. *R_f_* (*n*-hexane/acetone/triethylamine 1:1:0.01): 0.42.

^1^H NMR (300 MHz, DMSO-d_6_): *δ* 1.94 (m_c_, 2 H). 2.92–2.78 (m, 2 H), 3.95 (t, ^3^*J* = 6.9 Hz, 2 H), 7.02 (d, ^3^*J* = 8.9 Hz, 2 H), 7.47–7.35 (m, 4 H), 7.93 (s, 2 H). ^13^C NMR (151 MHz, DMSO-d_6_): *δ* 24.3 (CH_2_), 36.5 (CH_2_), 43.9 (CH_2_), 114.4 (C_quat_), 117.9 (CH), 125.9 (C_quat_), 129.2 (CH), 130.4 (CH), 143.6 (C_quat_). MS (EI) *m*/*z* (%): 416 (36, [M^81^Br_2_]^+^), 414 (70, [M^81^Br^79^Br]^+^), 412 (34, [M^79^Br_2_]^+^), 398 (20, [C_15_H_12_^81^Br_2_N^32^S]^+^), 396 (39, [C_15_H_12_^81^Br^79^Br N^32^S]^+^), 394 (19, [C_15_H_12_^79^Br_2_N^32^S]^+^), 372 (12, [C_13_H_8_^81^Br_2_N^32^S]^+^), 370 (24, [C_13_H_8_^81^Br^79^BrN^32^S]^+^), 368 (12, [C_13_H_8_^79^Br_2_N^32^S]^+^), 358 (48, [C_12_H_6_^81^Br_2_N^32^S]^+^), 356 (85, [C_12_H_6_^81^Br^79^BrN^32^S]^+^), 354 (42, [C_12_H_6_^79^Br_2_N^32^S]^+^), 291 (21, [C_13_H_8_^81^BrN^32^S]^+^), 289 (20, [C_13_H_8_^79^BrN^32^S]^+^), 277 (29, [C_12_H_6_^81^BrN^32^S]^+^), 196 (73, [C_12_H_6_N^32^S]^+^). IR $\tilde{\nu }$ [cm^−1^]: 3092 (vw), 3057 (vw), 2934 (vw), 1585 (w), 1479 (m), 1456 (s), 1387 (w), 1327 (w), 1294 (w), 1252 (m), 1229 (m), 1150 (w), 1109 (w), 1082 (w), 1057 (w), 868 (w), 806 (m), 750 (m), 650 (w).

#### 3.3.4. *tert*-Butyl (3-(3,7-Dibromo-10*H*-phenothiazin-10-yl)propyl)carbamate (**5**)

In a dry Schlenk tube with a magnetic stir bar, 3-(3,7-dibromo-10*H*-phenothiazin-10-yl)propylamine (**4**) (3.97 g, 8.80 mmol), di-*tert*-butyl dicarbonate (1.92 g, 8. 80 mmol), and 4-dimethylaminopyridine (108 mg, 0.88 mmol) were placed under nitrogen, and dichloromethane (44 mL) and triethylamine (2.45 mL, 17.7 mmol) were added. The reaction mixture was stirred at room temp for 16 h. The reaction was monitored by thin-layer chromatography. Then, the solvent was removed under reduced pressure, and the crude product was adsorbed on Celite^®^ and purified by column chromatography on silica gel (*n*-hexanes/acetone 9:1 to 7:3) to give *tert*-butyl (3-(3,7-dibromo-10*H*-phenothiazin-10-yl)propyl)carbamate (**5**) (4.30 g, 95%) as a beige-colored, glassy solid, Mp 70–72 °C. *R_f_* (*n*-hexane/acetone/triethylamine 7:3:0.1): 0.39.

^1^H NMR (300 MHz, DMSO-d_6_): *δ* 1.35 (s, 9 H), 1.76 (m, 2 H), 3.01 (m, 2 H), 3.83 (t, ^3^*J* = 6.5 Hz, 2 H), 6.86 (t, ^3^*J* = 4.9 Hz, 1 H), 6.96 (d, ^3^*J* = 7.7 Hz, 2 H), 7.38–7.33 (m, 4 H). ^13^C NMR (75 MHz, DMSO-d_6_): *δ* 26.4 (CH_2_), 28.2 (CH_3_), 37.6 (CH_2_), 44.5 (CH_2_), 77.5 (C_quat_), 114.1 (C_quat_), 117.6 (CH), 125.6 (C_quat_), 129.1 (CH), 130.3 (CH), 143.8 (C_quat_), 155.6 (C_quat_). MS (EI) *m*/*z* (%): 516 (14, [M^81^Br_2_]^+^), 514 (32, [M^81^Br^79^Br]^+^), 512 (14, [M^79^Br_2_]^+^), 460 (13, [C_16_H_14_^81^Br_2_N_2_O_2_^32^S]^+^), 458 (29, [C_16_H_14_^81^Br^79^Br N_2_O_2_^32^S]^+^), 456 (13, [C_16_H_14_^79^Br_2_N_2_O_2_^32^S]^+^), 357 (62, [C_12_H_7_^81^Br^79^Br N^32^S]^+^), 277 (30, [C_12_H_7_^79^BrN^32^S]^+^), 196 (39, [C_12_H_6_N^32^S]^+^), 102 (100, [C_5_H_9_O_2_]^+^). IR $\tilde{\nu }$ [cm^−1^]: 3395 (vw), 3379 (vw), 2976 (w), 2926 (w), 1694 (m), 1686 (m), 1516 (w), 1506 (m), 1481 (w), 1452 (vs), 1389 (m), 1364 (m), 1327 (w), 1267 (m), 1248 (s), 1202 (w), 1163 (s), 1109 (w), 1082 (w), 1040 (vw), 1003 (w), 959 (vw), 932 (vw), 868 (m), 802 (s), 779 (w), 750 (m), 652 (w). Anal. calcd. for C_20_H_22_Br_2_N_2_O_2_S [514.3]: C 46.71, H 4.31, N 5.45, S 6.23; Found: C 46.98, H 4.17, N 5.32, S 6.23.

### 3.4. General Procedure (GP1) for the Suzuki Synthesis of Boc-Protected 3,7-Di(hetero)aryl-substituted 10-(3-aminopropyl) 10H-Phenothiazines ***7***

Phenothiazine dibromide **5** (1.00 equiv), boronic acid or boronic acid ester **6** (2.20–2.40 equivs), cesium fluoride (6.40 equivs), and tetrakis(triphenylphosphane)palladium(0) (6.00 mol%) were placed in a dry Schlenk tube with a magnetic stir bar under nitrogen atmosphere, and 1,4-dioxane was added (for experimental details, see Table 3). The reaction mixture was heated to reflux at 101 °C for 16 h to give a yellow solution or suspension. After cooling to room temp, dichloromethane (20 to 90 mL) was added, and the solution was washed with distilled water (2 × 20 to 100 mL) and with saturated brine (1 × 20 to 100 mL). The aqueous phases were again extracted with dichloromethane. The combined organic phases were dried (anhydrous magnesium sulfate), and the crude product was adsorbed on Celite^®^ and purified by column chromatography on silica gel to give 3,7-di(hetero)aryl-substituted 10-(3-aminopropyl)-10*H*-phenothiazines **7**.

#### 3.4.1. *tert*-Butyl (3-(10,10″-Dihexyl-10*H*,10′*H*,10″*H*-[3,3′:7′,3″-terphenothiazin]-10′-yl)propyl)carbamate (**7a**)

According to the GP1 and after flash chromatography on silica gel (*n*-hexane/acetone 8:2), compound **7a** (1.56 g, 85%) was obtained as yellow crystals, Mp 98–104 °C. *R_f_* (*n*-hexane/acetone 8:2): 0.37.

^1^H NMR (300 MHz, acetone-d_6_): *δ* 0.85 (t, ^3^*J* = 7.1 Hz, 6 H), 1.23–1.35 (m, 8 H), 1.38 (s, 9 H), 1.47 (m_c_, 4 H), 1.81 (m_c_, 4 H), 2.03 (m_c_, 2 H), 3.26 (m_c_, 2 H), 3.95 (t, ^3^*J* = 7.0 Hz, 4 H), 4.05 (t, ^3^*J* = 7.0 Hz, 2 H), 6.10 (t, ^3^*J* = 5.8 Hz, 1 H), 6.90–6.98 (m, 2 H), 7.00–7.11 (m, 6 H), 7.13–7.23 (m, 4 H), 7.39 (m_c_, 4 H), 7.77 (m_c_, 4 H). ^13^C NMR (75 MHz, acetone-d_6_): *δ* 14.3 (CH_3_), 23.3 (CH_2_), 27.2 (CH_2_), 27.6 (CH_2_), 28.2 (CH_2_), 28.6 (CH_3_), 32.2 (CH_2_), 39.0 (CH_2_), 45.7 (CH_2_), 47.8 (CH_2_), 78.5 (C_quat_), 116.6 (CH), 116.8 (CH), 116.8 (CH), 123.3 (CH), 125.1 (C_quat_), 125.5 (CH), 125.6 (CH), 125.7 (C_quat_), 126.0 (C_quat_), 126.1 (CH), 126.2 (CH), 128.0 (CH), 128.3 (CH), 134.9 (C_quat_), 135.1 (C_quat_), 144.9 (C_quat_), 145.3 (C_quat_), 146.0 (C_quat_), 156.8 (C_quat_). MS (MALDI-TOF) *m*/*z* calcd. For [C_56_H_62_N_4_O_2_S_3_]^+^: 918.403; Found: 918.431. IR $\tilde{\nu }$ [cm^−1^]: 2957 (m), 2926 (m), 2901 (w), 1709 (m), 1601 (w), 1576 (w), 1499 (m), 1456 (vs), 1414 (w), 1377 (m), 1364 (m), 1331 (m), 1240 (s), 1163 (m), 1140 (m), 1105 (w), 1065 (w), 1042 (w), 1011 (vw), 874 (m), 854 (vw), 806 (s), 779 (w), 745 (s), 706 (vw), 623 (w), 606 (m). Anal. Calcd. For C_56_H_62_N_4_O_2_S_3_ [919.3]: C 73.16, H 6.80, N 6.09, S 10.46; Found: C 73.30, H 6.76, N 5.89, S 10.25.

#### 3.4.2. *tert*-Butyl (3-(3,7-Bis(4-methoxyphenyl)-10*H*-phenothiazin-10-yl)propyl)carbamate (**7b**)

According to the GP1 and after flash chromatography on silica gel (*n*-hexane/acetone 8:2), compound **7b** (630 mg, 79%) was obtained as a colorless solid, Mp 151–156 °C. *R_f_* (*n*-hexane/acetone 8:2): 0.31. 

^1^H NMR (300 MHz, DMSO-d_6_): *δ* 1.36 (s, 9 H), 1.86 (m_c_, 2 H), 3.08 (m_c_, 2 H), 3.77 (s, 6 H), 3.91 (t, ^3^*J* = 6.9 Hz, 2 H), 6.92 (t, ^3^*J* = 5.4 Hz, 1 H), 6.98 (d, ^3^*J* = 8.9 Hz, 4 H), 7.06 (d, ^3^*J* = 8.4 Hz, 2 H), 7.39 (d, ^4^*J* = 2.2 Hz, 2 H), 7.43 (dd, ^3^*J* = 8.4 Hz, ^4^*J* = 2.2 Hz, 2 H), 7.56 (d, ^3^*J* = 8.9 Hz, 4 H). ^13^C NMR (75 MHz, DMSO-d_6_): *δ* 26.8 (CH_2_), 28.3 (CH_3_), 44.5 (CH_2_), 55.2 (CH_3_), 77.7 (C_quat_), 114.4 (CH), 115.9 (CH), 123.8 (C_quat_), 124.6 (CH), 125.3 (CH), 127.2 (CH), 131.4 (C_quat_), 134.3 (C_quat_), 143.2 (C_quat_), 155.8 (C_quat_), 158.7 (C_quat_). MS (EI) *m*/*z* (%): 568 (40, [M]^+^), 512 (42, [C_30_H_27_N_2_O_4_S]^+^), 424 (9, [C_27_H_22_NO_2_S]^+^), 410 (100, [C_26_H_20_NO_2_S]^+^), 414 (15, [C_27_H_16_N_3_S]^+^), 400 (100, [C_26_H_14_N_3_S]^+^). IR $\tilde{\nu }$ [cm^−1^]: 2970 (w), 2951 (w), 2930 (w), 2868 (vw), 2833 (w), 1697 (s), 1682 (m), 1607 (m), 1580 (w), 1518 (m), 1493 (m), 1460 (s), 1439 (m), 1430 (m), 1391 (m), 1364 (m), 1331 (m), 1281 (m), 1242 (vs), 1209 (m), 1179 (s), 1113 (m), 1086 (w), 1047 (m), 1024 (m), 1009 (m), 955 (w), 926 (w), 912 (w), 883 (w), 808 (s), 775 (m), 768 (m), 741 (w), 729 (w). 714 (w), 687 (w), 671 (m). Anal. calcd. for C_34_H_30_N_4_O_2_S [568.7]: C: 71.80, H: 6.38, N: 4.93, S: 5.64, Found: C: 71.69, H: 6.49, N: 4.68, S: 5.48.

#### 3.4.3. *tert*-Butyl (3-(3,7-Di(thien-2-yl)-10*H*-phenothiazin-10-yl)propyl)carbamate (**7c**) [16]

According to the GP1 and after flash chromatography on silica gel (*n*-hexane/acetone 8:2), compound **7c** (359 mg, 69%) was obtained as a yellow powder, Mp 138–141 °C. *R_f_* (*n*-hexane/acetone 7:3): 0.34. 

^1^H NMR (600 MHz, DMSO-d_6_): *δ* 1.36 (s, 9 H), 1.84 (m_c_, 2 H), 3.07 (m_c_, 2 H), 3.91 (t, ^3^*J* = 7.0 Hz, 2 H), 6.92, (t, ^3^*J* = 5.5 Hz, 1 H), 7.04 (d, ^3^*J* = 9.1 Hz, 2 H), 7.04 (dd, ^3^*J* = 9.1 Hz, 2 H), 7.46–7.42 (m, 6 H), 7.48 (dd, ^3^*J* = 5.1 Hz, ^4^*J* = 1.1 Hz, 2 H). ^13^C NMR (151 MHz, DMSO-d_6_): *δ* 26.6 (CH_2_), 28.3 (CH_3_), 37.7 (CH_2_), 44.5 (CH_2_), 77.5 (C_quat_), 116.1 (CH), 123.1 (CH), 123.6 (C_quat_), 123.7 (CH), 124.9 (CH), 125.0 (CH), 128.5 (CH), 128.5 (C_quat_), 142.2 (C_quat_), 143.4 (C_quat_), 155.6 (C_quat_). MS (EI) *m*/*z* (%): 520 (32, [C_28_H_28_N_2_O_2_S_3_]^+^), 464 (24, [C_24_H_19_N_2_O_2_S_3_]^+^), 446 (6, [C_24_H_19_N_2_OS_3_]^+^), 420 (13, [C_23_H_19_N_2_S_3_]^+^), 376 (8, [C_21_H_14_NS_3_]^+^), 362 (100, [C_20_H_12_NS_3_]^+^). IR $\tilde{\nu }$ [cm^−1^]: 2974 (w), 2926 (w), 2864 (w), 1701 (m), 1680 (m), 1472 (vs), 1429 (m), 1400 (m), 1391 (m), 1364 (m), 1348 (w), 1333 (w), 1292 (w), 1261 (s), 1238 (s), 1207 (m), 1163 (s), 1109 (m), 1080 (w), 1042 (w), 1020 (w), 1007 (vw), 988 (w), 951 (vw), 951 (vw), 874 (m), 851 (m), 810 (s), 793 (m), 783 (w), 750 (m), 691 (vs), 650 (vw). Anal. Calcd. For C_28_H_28_N_2_O_2_S_3_ [519.7]: C 64.58, H 5.42, N 5.38, S 18.47; Found: C 64.83, H 5.69, N 5.12, S 18.17.

#### 3.4.4. *tert*-Butyl (3-(3,7-Diphenyl-10*H*-phenothiazin-10-yl)propyl)carbamate (**7d**)

According to the GP1 and after flash chromatography on silica gel (*n*-hexane/acetone 8:2), compound **7d** (560 mg, 74%) was obtained as yellow crystals, Mp 92–98 °C. *R_f_* (*n*-hexane/acetone 8:2): 0.39. 

^1^H NMR (300 MHz, DMSO-d_6_): *δ* 1.37 (s, 9 H), 1.88 (m_c_, 2 H), 3.09 (m_c_, 2 H), 3.95 (t, ^3^*J* = 6.9 Hz, 2 H), 6.94 (t, ^3^*J* = 5.3 Hz, 1 H), 7.11 (d, ^3^*J* = 8.4 Hz, 2 H), 7.29–7.36 (m, 2 H). 7.39–7.48 (m, 6 H), 7.50 (dd, ^3^*J* = 8.4 Hz, ^4^*J* = 2.2 Hz, 2 H), 7.60–7.66 (m, 4 H). ^13^C NMR (75 MHz, DMSO-d_6_): *δ* 26.7 (CH_2_), 28.3 (CH_3_), 37.8 (CH_2_), 44.5 (CH_2_), 77.5 (C_quat_), 116.0 (CH), 123.7 (C_quat_), 125.0 (CH), 125.8 (CH), 126.1 (CH), 127.1 (CH), 128.9 (CH), 134.5 (C_quat_), 138.9 (C_quat_), 143.7 (C_quat_), 155.7 (C_quat_). MS (EI) *m*/*z* (%): 508 (36, [M]^+^), 452 (32, [C_28_H_23_N_2_O_2_S]), 408 (4, [C_27_H_23_N_2_S]), 364 (12, [C_25_H_18_NS]), 350 (100, [C_24_H_16_NS]). IR $\tilde{\nu }$ [cm^−1^]: 2968 (w), 2901 (w), 2887 (w), 2870 (w), 1738 (w), 1678 (s), 1599 (w), 1585 (w), 1526 (m), 1462 (s), 1393 (m), 1364 (m), 1329 (w), 1275 (s), 1248 (s), 1202 (w), 1165 (s), 1157 (s), 1111 (m), 1078 (m), 1055 (m), 1042 (w), 1023 (m), 1003 (m), 986 (w), 939 (vw), 876 (m), 868 (m), 853 (w), 820 (m), 812 (m), 762 (vs), 737 (m), 700 (s), 689 (m), 613 (m). Anal. calcd. for C_32_H_32_N_2_O_2_S [508.7]: C 75.56, H 6.34, N 5.51, S 5.92; Found: C 75.20, H 6.40, N 5.12, S 5.92.

#### 3.4.5. *tert*-Butyl (3-(3,7-Bis(4-chlorophenyl)-10*H*-phenothiazin-10-yl)propyl)carbamate (**7e**)

According to the GP1 and after flash chromatography on silica gel (*n*-hexane/acetone 8:2), compound **7e** (660 mg, 76%) was obtained as yellow crystals, Mp 95–102 °C. *R_f_* (*n*-hexane/acetone 8:2): 0.38. 

^1^H NMR (600 MHz, acetone-d_6_): *δ* 1.38 (s, 9 H), 3.27 (m_c_, 2 H), 4.07 (t, ^3^*J* = 6.8 Hz, 2 H), 6.12 (t, ^3^*J* = 5.3 Hz, 1 H), 7.13 (m_c_, 2 H), 7.42–7.47 (m, 6 H), 7.49 (d, ^3^*J* = 8.4 Hz, 2 H), 7.64 (d, ^3^*J* = 7.8 Hz, 4 H). ^13^C NMR (151 MHz, acetone-d_6_): *δ* 28.1 (CH_2_), 28.6 (CH_3_), 39.0 (CH_2_), 45.8 (CH_2_), 78.6 (C_quat_), 116.9 (CH), 125.7 (C_quat_), 126.1 (CH), 126.8 (CH), 128.7 (CH), 129.7 (CH), 133.4 (C_quat_), 134.9 (C_quat_), 139.3 (C_quat_), 145.4 (C_quat_), 156.8 (C_quat_). MS (EI) *m*/*z* (%): 576 (40, [M]^+^), 520 (46, [C_28_H_21_Cl_2_N_2_O_2_S]), 504 (6, [C_28_H_21_Cl_2_N_2_OS]), 476 (3, [C_27_H_21_Cl_2_N_2_S]), 432 (14, [C_25_H_16_Cl_2_NS]), 419 (100, [C_24_H_14_Cl_2_NS]). IR $\tilde{\nu }$ [cm^−1^]: 3898 (vw), 3834 (vw), 3688 (vw), 3381 (vw), 3354 (vw), 3032 (vw), 2934 (vw), 2845 (vw), 2818 (vw), 2357 (vw), 1697 (m), 1684 (m), 1607 (w), 1585 (w), 1506 (m), 1460 (vs), 1418 (w), 1387 (m), 1364 (m), 1339 (w), 1271 (m), 1248 (s), 1200 (m), 1163 (s), 1092 (m), 1011 (m), 1003 (m), 959 (w), 910 (w), 885 (w), 876 (w), 808 (vs), 779 (m), 764 (m), 723 (w), 635 (w). Anal. Calcd. For C_32_H_30_Cl_2_N_2_O_2_S [577.6]: C 66.55, H 5.24, N 4.85, S 5.55; Found: C 66.83, H 5.51, N 4.56, S 5.54.

#### 3.4.6. *tert*-Butyl (3-(3,7-Bis(4-cyanophenyl)-10*H*-phenothiazin-10-yl)propyl)carbamate (**7f**)

According to the GP1 and after flash chromatography on silica gel (*n*-hexane/acetone 7:3), compound **7f** (530 mg, 67%) was obtained as a yellow powder, Mp 183–185 °C. *R_f_* (*n*-hexane/acetone 8:2): 0.22. ^1^H NMR (300 MHz, acetone-d_6_): *δ* 1.38 (s, 9 H), 2.07 (m, 2 H), 3.25–3.31 (m, 2 H), 4.11 (t, ^3^*J* = 7.0 Hz, 2 H), 6.13 (t, ^3^*J* = 5.3 Hz, 1 H), 7.19 (d, ^3^*J* = 8.6 Hz, 2 H), 7.55 (d, ^4^*J* = 2.1 Hz, 2 H), 7.60 (dd, ^3^*J* = 8.6 Hz, ^4^*J* = 2.1 Hz, 2 H), 7.82 (d, ^3^*J* = 8.4 Hz, 4 H), 7.86 (d, ^3^*J* = 8.4 Hz, 4 H). ^13^C NMR (75 MHz, acetone-d_6_): *δ* 28.1 (CH_2_), 28.6 (CH_3_), 38.9 (CH_2_), 45.9 (CH_2_), 78.6 (C_quat_), 111.3 (C_quat_), 117.1 (CH), 119.4 (C_quat_), 125.7 (C_quat_), 126.5 (CH), 127.4 (CH), 127.9 (CH), 133.5 (CH), 134.3 (C_quat_), 144.8 (C_quat_), 146.0 (C_quat_), 156.8 (C_quat_). MS (EI) *m*/*z* (%): 558 (1, [M]^+^), 484 (50, [C_30_H_22_N_4_OS]^+^), 414 (15, [C_27_H_16_N_3_S]^+^), 400 (100, [C_26_H_14_N_3_S]^+^). IR $\tilde{\nu }$ [cm^−1^]: 3393 (vw), 3364 (w), 2974 (w), 2932 (w), 2901 (vw), 2870 (vw), 2220 (s), 1676 (s), 1605 (m), 1585 (m), 1526 (m), 1518 (m), 1477 (vs), 1445 (w), 1418 (m), 1393 (s), 1364 (s), 1271 (s), 1244 (s), 1163 (s), 1125 (m), 1115 (m), 1038 (w), 1001 (m), 833 (s), 793 (vs), 648 (m). Anal. calcd. for C_34_H_30_N_4_O_2_S [558.7]: C: 73.09, H: 5.41, N: 10.03, S: 5.74, Found: C: 72.86, H: 5.50, N: 9.78, S: 5.71.

### 3.5. General Procedure (GP2) for the Synthesis of 3,7-Di(hetero)aryl-substituted 10-(3-Trimethylammoniumpropyl)10H-phenothiazine Salts ***8***

In a Schlenk flask with a magnetic stir bar, Boc-protected 3,7-di(hetero)aryl-substituted 10-(3-aminopropyl) 10*H*-phenothiazine **7** (1.00 equiv) was dissolved in dichloromethane (10.0 mL/mmol) under nitrogen atmosphere, and trifluoroacetic acid (8.00 equivs) was added, whereby the color immediately changed from yellow to green-brown (for experimental details, see Table 4). The reaction mixture was stirred at room temp for 16 h, after which the volatile components were removed under reduced pressure. The residue was dissolved in a 1:1 mixture of methanol and dichloromethane (10.0 mL/mmol), and potassium carbonate (20.0 equivs) was slowly added. Methyl iodide or methyl triflate (10.0 equivs) was added dropwise to the reaction mixture. The reaction solution was stirred at room temp for 16 h. The crude product was adsorbed on neutral alumina and purified by column chromatography on neutral alumina to give the trimethylammonium salts **8**.

#### 3.5.1. Bis{3-(10,10″-dihexyl-10*H*,10′*H*,10″*H*-[3,3′:7′,3″-terphenothiazin]-10′-yl)-*N*,*N*,*N*-trimethylpropan-1-ammonium} Carbonate (**8a**)

According to the GP2 and after flash chromatography on neutral alumina (dichloromethane/methanol 100:0 to 100:1), compound **8a** (823 mg, 93%) was obtained as yellow crystals, Mp softening at 127 °C, melting between 148 and 152 °C. *R_f_* (dichloromethane/methanol 100:1): 0.17.

^1^H NMR (600 MHz, DMSO-d_6_): *δ* 0.82 (t, ^3^*J* = 6.9 Hz, 6 H), 1.21–1.29 (m, 8 H), 1.39 (m_c_, 4 H), 1.69 (m_c_, 4 H), 2.15 (m_c_, 2 H), 3.04 (s, 9 H), 3.44 (m_c_, 2 H), 3.88 (t, ^3^*J* = 6.9 Hz, 4 H), 3.99 (t, ^3^*J* = 6.9 Hz, 2 H), 6.92–6.97 (m, 2 H), 7.00–7.06 (m, 4 H), 7.13 (d, ^3^*J* = 8.6 Hz, 2 H), 7.15 (dd, ^3^*J* = 7.6 Hz, ^4^*J* = 1.3 Hz, 2 H), 7.18–7.22 (m, 4 H), 7.42 (d, ^4^*J* = 2.2 Hz, 2 H), 7.46–7.51 (m, 4 H). ^13^C NMR (151 MHz, acetone-d_6_): *δ* 14.3 (CH_3_), 21.6 (CH_2_), 23.3 (CH_2_), 27.2 (CH_2_), 27.5 (CH_2_), 32.2 (CH_2_), 44.6 (CH_2_), 47.8 (CH_2_), 53.8 (CH_3_), 64.8 (CH_2_), 116.6 (CH), 116.8 (CH), 117.3 (CH), 123.3 (CH), 125.1 (C_quat_), 125.5 (CH), 125.6 (CH), 125.8 (C_quat_), 126.0 (C_quat_), 126.2 (CH), 126.5 (CH), 128.0 (CH), 128.4 (CH), 134.7 (C_quat_), 135.3 (C_quat_), 144.3 (C_quat_), 145.3 (C_quat_), 146.0 (C_quat_). MS (MALDI-TOF) *m*/*z* calcd. for [C_54_H_61_N_4_S_3_]^+^: 861.405; Found: 861.408. IR $\tilde{\nu }$ [cm^−1^]: 2951 (w), 2924 (w), 2899 (w), 2866 (w), 2853 (w), 1601 (w), 1574 (w), 1454 (vs) 1416 (m), 1373 (m), 1331 (m), 1275 (w), 1238 (s), 1196 (m), 1134 (w), 1105 (m), 1078 (w), 1065 (w), 1040 (w), 1028 (w), 964 (w), 908 (w), 872 (w), 808 (m), 745 (s), 704 (w), 683 (w), 650 (w), 631 (w). Anal. calcd. for (C_54_H_61_N_4_S_3_)_2_CO_3_ [1784.6]: C 73.36, H 6.89, N 6.28, S 10.78; Found: C 73.23, H 6.83, N 5.91, S 10.41.

#### 3.5.2. 3-(3,7-Bis(4-methoxyphenyl)-10*H*-phenothiazin-10-yl)-*N*,*N*,*N*-trimethylpropan-1-ammoniumtriflate (**8b**)

According to the GP2 and after flash chromatography on neutral alumina (dichloromethane/methanol 100:1 to 100:2), compound **8b** (260 mg, 67%) was obtained as a pale beige powder, Mp 200–205 °C. *R_f_* (*n*-hexane/acetone 4:6): 0.11.

^1^H NMR (300 MHz, acetone-d_6_): *δ* 2.50 (m_c_, 2 H), 3.79 (m_c_, 2 H), 3.34 (s, 9 H), 3.83 (s, 6 H), 4.21 (t, ^3^*J* = 6.7 Hz, 2 H), 7.00 (d, ^3^*J* = 8.8 Hz, 4 H), 7.17 (d, ^3^*J* = 8.3 Hz, 2 H), 7.44–7.51 (m, 4 H), 7.56 (d, ^3^*J* = 8.8 Hz, 4 H). ^13^C NMR (75 MHz, acetone-d_6_): *δ* 21.9 (CH_2_), 44.8 (CH_2_), 53.9 (CH_3_), 53.9 (CH_3_), 54.0 (CH_3_), 55.7 (CH_3_), 65.5 (CH_2_), 115.3 (CH), 115.5 (C_quat_), 117.2 (CH), 126.0 (CH), 126.6 (CH), 128.3 (CH), 131.6 (C_quat_), 132.9 (C_quat_), 136.7 (C_quat_), 144.4 (C_quat_), 160.4 (C_quat_). MS (ESI) *m*/*z* calcd. for [C_32_H_35_N_2_O_2_S]^+^: 511.2; Found: 511.5. IR $\tilde{\nu }$ [cm^−1^]: 2930 (vw), 1609 (m), 1518 (w), 1491 (m), 1464 (s), 1443 (m), 1423 (w), 1393 (w), 1341 (w), 1238 (vs), 1225 (s), 1198 (m), 1177 (s), 1155 (s), 1111 (m), 1082 (m), 1045 (m), 1028 (vs), 964 (w), 937 (vw), 910 (m), 883 (w), 808 (s), 773 (w), 756 (m), 637 (vs). Anal. calcd. for C_33_H_35_F_3_N_2_O_5_S_2_ [660.8]: C 59.99, H 5.34, N 4.24, S 9.70; Found: C 60.24, H 5.39, N 4.05, S 9.47.

#### 3.5.3. 3-(3,7-Di(thiophen-2-yl)-10*H*-phenothiazin-10-yl)-*N*,*N*,*N*-trimethylpropan-1-ammoniumtriflate (**8c**)

According to the GP2 and after flash chromatography on neutral alumina (dichloromethane/methanol 100:1 to 100:2), compound **8c** (140 mg, 59%) was obtained as a yellow powder, Mp 128–130 °C. *R_f_* (methanol): 0.10.

^1^H NMR (600 MHz, DMSO-d_6_): *δ* 2.50 (m_c_, 2 H), 3.80 (m_c_, 2 H), 4.21 (t, ^3^*J* = 6.9 Hz, 2 H), 7.04 (dd, ^3^*J* = 3.6 Hz, ^4^*J* = 1.1 Hz, 2 H), 7.11 (m_c_, 2 H), 7.16 (d, ^3^*J* = 8.3 Hz, 2 H), 7.43 (dd, ^3^*J* = 5.1 Hz, ^4^*J* = 0.9 Hz, 2 H), 7.52–7.49 (m, 4 H). ^13^C NMR (125 MHz, methanol-d_4_): *δ* 22.0 (CH_2_), 45.0 (CH_2_), 53.7 (CH_3_), 65.8 (CH_2_), 117.6 (CH), 123.9 (CH), 125.6 (CH), 125.6 (CH), 126.4 (CH), 127.5 (C_quat_), 129.2 (CH), 131.5 (C_quat_), 144.1 (C_quat_), 145.1 (C_quat_). IR $\tilde{\nu }$ [cm^−1^]: 3034 (vw), 2963 (w), 1603 (vw), 1472 (s), 1427 (m), 1402 (m), 1346 (w), 1339 (w), 1258 (vs), 1223 (m), 1200 (w), 1153 (m), 1084 (s), 1045 (s), 1028 (vs), 1015 (s), 966 (w), 943 (vw), 910 (w), 872 (m), 851 (m), 799 (vs), 752 (m), 737 (w), 692 (s), 662 (vw), 637 (s), 610 (w). ESI-HRMS *m*/*z* calcd. for [C_26_H_27_N_2_S_3_]^+^: 463.1331; Found: 463.1336.

#### 3.5.4. 3-(3,7-Diphenyl-10*H*-phenothiazin-10-yl)-*N*,*N*,*N*-trimethylpropan-1-ammoniumtriflate (**8d**)

According to the GP2 and after flash chromatography on neutral alumina (dichloromethane/methanol 100:1 to 100:2), compound **8d** (240 mg, 81%) was obtained as yellow crystals, Mp softening at 110 °C, melting between 119 and 129 °C. *R_f_* (*n*-hexane/acetone 4:6): 0.13.

^1^H NMR (600 MHz, DMSO-d_6_): *δ* 2.18 (m_c_, 2 H), 3.05 (s, 9 H), 3.46 (m_c_, 2 H), 4.02 (t, ^3^*J* = 6.9 Hz, 2 H), 7.19 (d, ^3^*J* = 8.5 Hz, 2 H), 7.33 (m_c_, 2 H), 7.44 (m_c_, 4 H), 7.52 (d, ^4^*J* = 2.1 Hz, 2 H), 7.55 (dd, ^3^*J* = 8.5 Hz, ^4^*J* = 2.1 Hz, 2 H), 7.64 (d, ^3^*J* = 7.5 Hz, 4 H). ^13^C NMR (151 MHz, DMSO-d_6_): *δ* 20.3 (CH_2_), 43.7 (CH_2_), 52.4 (CH_3_), 63.2 (CH_2_), 116.2 (CH), 124.1 (C_quat_), 125.2 (CH), 126.0 (CH), 126.1 (CH), 127.3 (CH), 129.0 (CH), 134.9 (C_quat_), 138.8 (C_quat_), 143.4 (C_quat_). MS (MALDI-TOF) *m*/*z* calcd. for [C_31_H_31_F_3_N_2_O_3_S_2_]^+^: 451.220; Found: 451.325. IR $\tilde{\nu }$ [cm^−1^]: 1599 (w), 1464 (s), 1393 (w), 1329 (w), 1252 (s), 1223 (m), 1196 (w), 1152 (m), 1109 (w), 1074 (w), 1028 (s), 966 (w), 935 (w), 914 (w), 883 (w), 826 (w), 760 (s), 737 (w), 696 (s), 637 (vs), 629 (m), 608 (m). Anal. calcd. for C_31_H_31_F_3_N_2_O_3_S_2_ [600.7]: C 61.98, H 5.20, N 4.66, S 10.67; Found: C 62.23, H 5.30, N 4.59, S 10.38.

#### 3.5.5. 3-(3,7-Bis(4-chlorophenyl)-10*H*-phenothiazin-10-yl)-*N*,*N*,*N*-trimethylpropan-1-ammoniumtriflate (**8e**)

According to the GP2 and after flash chromatography on neutral alumina (dichloromethane/methanol 100:1 to 100:2), compound **8e** (210 mg, 71%) was obtained as a greenish powder, Mp softening at 151 °C, melting between 181 and 192 °C. *R_f_* (*n*-hexane/acetone 4:6): 0.10.

^1^H NMR (600 MHz, acetone-d_6_): *δ* 2.49 (m_c_, 2 H), 3.34 (s, 9 H), 3.82 (m_c_, 2 H), 4.22 (t, ^3^*J* = 6.9 Hz), 7.24 (d, ^3^*J* = 8.5 Hz, 2 H), 7.45 (d, ^3^*J* = 8.6 Hz, 4 H), 7.50 (d, ^4^*J* = 2.2 Hz, 2 H), 7.55 (dd, ^3^*J* = 8.5 Hz, ^4^*J* = 2.2 Hz, 2 H), 7.65 (d, ^3^*J* = 8.6 Hz, 4 H). ^13^C NMR (151 MHz, acetone-d_6_): *δ* 21.6 (CH_2_), 44.7 (CH_2_), 53.7 (CH_3_), 53.7 (CH_3_), 53.8 (CH_3_), 65.0 (CH_2_), 117.3 (CH), 126.1 (C_quat_), 126.2 (CH), 127.1 (CH), 128.8 (CH), 129.8 (CH), 133.5 (C_quat_), 135.3 (C_quat_), 139.1 (C_quat_), 145.0 (C_quat_). MS (MALDI-TOF) *m*/*z* calcd. for [C_30_H_29_^35^Cl_2_N_2_S]^+^: 519.142; Found: 519.148. IR $\tilde{\nu }$ [cm^−1^]: 3028 (vw), 2857 (vw), 1460 (s), 1414 (w), 1383 (w), 1341 (w), 1317 (vw), 1254 (s), 1225 (m), 1198 (w), 1157 (s), 1105 (w), 1092 (s), 1055 (w), 1028 (s), 1011 (m), 962 (w), 939 (vw), 910 (w), 891 (w), 810 (m), 791 (m), 768 (w), 756 (w), 745 (w), 723 (w), 691 (w), 637 (vs), 610 (w). Anal. calcd. for C_31_H_29_Cl_2_F_3_N_2_O_3_S_2_ [669.6]: C 55.61, H 4.37, N 4.18, S 9.58; Found: C 55.53, H 4.55, N 3.98, S 9.28.

#### 3.5.6. 3-(3,7-Bis(4-cyanophenyl)-10*H*-phenothiazin-10-yl)-*N*,*N*,*N*-trimethylpropan-1-ammoniumtriflate (**8f**)

According to the GP2 and after flash chromatography on neutral alumina (dichloromethane/methanol (100:1 to 100:2)), compound **8f** (150 mg, 53%) was obtained as a yellow powder, Mp softening at 145 °C, melting between 168 and 172 °C. *R_f_* (acetone): 0.08.

^1^H NMR (300 MHz, acetone-d_6_): *δ* 2.52 (m_c_, 2 H), 3.35 (s, 9 H), 3.82 (m_c_, 2 H), 4.26 (t, ^3^*J* = 6.9 Hz, 2 H), 7.29 (d, ^3^*J* = 8.5 Hz, 2 H), 7.61 (d, ^4^*J* = 2.2 Hz, 2 H), 7.66 (dd, ^3^*J* = 8.5 Hz, ^4^*J* = 2.2 Hz, 2 H), 7.85 (m_c_, 8 H). ^13^C NMR (151 MHz, acetone-d_6_): *δ* 21.9 (CH_2_), 44.9 (CH_2_), 53.9 (CH_3_), 54.0 (CH_3_), 54.0 (CH_3_), 65.3 (CH_2_), 111.6 (C_quat_), 117.5 (CH), 119.4 (C_quat_), 126.4 (C_quat_), 126.7 (CH), 127.7 (CH), 128.0 (CH), 133.6 (CH), 133.8 (C_quat_), 134.9 (C_quat_), 144.8 (C_quat_), 145.8 (C_quat_). MS (MALDI-TOF) *m*/*z* calcd. for [C_32_H_29_N_4_S]^+^: 501.211; Found: 501.222. IR $\tilde{\nu }$ [cm^−1^]: 2930 (w), 2855 (m), 2224 (w), 1730 (w), 1605 (m), 1585 (w), 1468 (s), 1420 (m), 1364 (m), 1344 (w), 1248 (s), 1225 (m), 1198 (m), 1155 (s), 1109 (s), 1055 (m), 1030 (s), 962 (m), 914 (m), 881 (m), 843 (m), 810 (s), 743 (m), 689 (m), 637 (vs). Anal. calcd. for C_33_H_29_F_3_N_4_O_3_S_2_ [650.7]: C 60.91, H 4.49, N 8.61, S 9.85; Found: C 60.25, H 4.84, N 8.17, S 8.56.

### 3.6. N,N,N-Trimethyl-3-(10H-phenothiazin-10-yl)propan-1-ammoniumtriflate *(**9**)* [16]

Compound **9** (332 mg, 90%) was synthesized according to [16] and obtained after purification as a colorless powder, Mp 168–170 °C. *R_f_* (methanol): 0.31.

^1^H NMR (300 MHz, methanol-d_4_): *δ* 2.25 (m_c_, 2 H). 3.01 (s, 9 H), 3.45 (m_c_, 2 H), 4.11 (t, ^3^*J* = 6.3 Hz, 2 H), 6.99 (m_c_, 2 H), 7.06 (dd, ^3^*J* = 8.1 Hz, ^4^*J* = 0.9 Hz, 2 H), 7.19 (dd, ^3^*J* = 7.6 Hz, ^4^*J* = 1.6 Hz, 2 H), 7.25 (m_c_, 2 H). ^13^C NMR (125 MHz, methanol-d_4_): *δ* 21.8 (CH_2_), 44.7 (CH_2_), 53.5 (CH_3_), 65.7 (CH_2_), 117.3 (CH), 124.3 (CH), 127.4 (C_quat_), 128.6 (CH), 128.8 (CH), 146.2 (C_quat_). IR $\tilde{\nu }$ [cm^−1^]: 3065 (vw), 3042 (vw), 2965 (vw), 2868 (vw), 1593 (w), 1570 (w), 1485 (m), 1479 (m), 1460 (s), 1445 (m), 1422 (w), 1400 (w), 1339 (w), 1254 (vs), 1221 (s), 1200 (w), 1157 (s), 1140 (s), 1128 (m), 1109 (w), 1028 (s), 961 (w), 939 (w), 918 (w), 908 (w), 764 (s), 754 (w), 729 (m), 637 (s). MS (EI) m/z (%): 448 (1, [M]^+^), 299 (10, [C_18_H_23_N_2_S]^+^) 284 (41, [C_17_H_20_N_2_S]^+^), 239 (100, [C_15_H_14_NS]^+^), 212 (18, [C_13_H_10_NS]^+^), 199 (50, [C_12_H_8_NS]^+^). Anal. calcd. for C_19_H_23_F_3_N_2_O_3_S_2_ [448.5]: C 50.88, H 5.17, N 6.25, S 14.30; Found: C 50.83, H 5.36, N 6.09, S 14.05.

## 4. Conclusions

Starting from 3,7-dibromo phenothiazine, a library of 3,7-di(hetero)aryl-substituted 10-(3-trimethylammoniumpropyl)10*H*-phenothiazine salts can be prepared in five steps. As is typical for phenothiazines, all derivatives are redox systems with fully reversible first oxidations. The Hammett correlation reveals that the remote polar substituents in the consanguineous series of 3,7-di(hetero)aryl-substituted phenothiazine salts and their precursors correlate very well with the σ_p_ parameters. All salts and precursors can be excited in UV with a substituent tunable wavelength, and the compounds display blue to green-blue emission. The screening of the selected 3,7-di(hetero)aryl-substituted 10-(3-trimethylammoniumpropyl)10*H*-phenothiazine salts discloses for some derivatives a distinct inhibition of the pathogenic bacterial strains *M. tuberculosis*, *S. aureus*, *E. coli*, *A. baumannii*, and *K. pneumoniae*; however, the distinct inhibition of *P. aeruginosa* is not disclosed. Based on the ease of the synthetic approach, further 3,7-diaryl- and 3-aryl-substituted phenothiazines with an *N*-propyl trimethylammonium sidechain can be tested; eventually, irradiation with UV light can also occur to trigger photoinduced electron transfer. Studies focused on scrutinizing the photophysical and biological properties of these *N*-propyl trimethylammonium phenothiazine derivatives are currently underway.

## Figures and Tables

**Table 1:** Selected electronic data (oxidation potential, absorption maxima, emission maxima, Stokes shifts) of Boc-protected 3,7-di(hetero)aryl-substituted 10-(3-aminopropyl) 10*H*-phenothiazines **7** and 3,7-di(hetero)aryl-substituted 10-(3-trimethylammoniumpropyl)10*H*-phenothiazine salts **8**.

Compound	*E*_0_^0/+1^ [V] [a]	*E*_0_^+1/+2^ [V] [a]	*E*_0_^+2/+3^ [V] [a]	Absorption *λ*_max,abs_ [nm] (*ε* [m^−1^cm^−1^]) [b]	Emission *λ*_max,em_ [nm] [c]	Stokes Shift ∆$\tilde{\nu }$ [cm^−1^] [d]
**7a**	0.620	0.740	0.850	279 (111,000), 320 (45,000), **367** (37,000sh)	483, 499 (sh)	6500
**7b**	0.640	1.290	-	280 (108,000), **330** (26,000)	461	9300
**7c**	0.690	1.260	-	290 (42,000), **347** (14,000)	488	8300
**7d**	0.700	1.400	-	277 (17,000), **330** (41,000)	471	9500
**7e**	0.730	-	-	280 (35,000), **333** (9000)	488	9500
**7f**	0.830	-	-	292 (33,000), **373** (12,000)	520	7600
**8a**	0.610	0.720	0.830	276 (83,000), 322 (31,000), **368** (26,000)	478	6300
**8b**	0.680	1.040	-	278 (64,000), **324** (15,000)	455	8900
**8c**	0.720	1.260	-	278 (40,000), **326** (10,000)	466	9200
**8d**	0.730	1.270	-	275 (26,000), **323** (6000)	456	9000
**8e**	0.770	-	-	289 (53,000), **342** (18,000)	478	8300
**8f**	0.840	-	-	288 (54,000), **359** (7000)	500	7900

[a] Cyclic voltammograms were recorded in dichloromethane at *T* = 293 K. [b] Recorded at *T* = 293 K in dichloromethane at *c*(**7**/**8**) = 10^−3^ m. [c] Recorded in dichloromethane at *T* = 293 K at *c*(**7**/**8**) = 10^−6^ m. [d] ∆$\tilde{\nu }$ = 1/*λ*_max,abs_ − 1/*λ*_max,em_ [cm^−1^] (calculated using the bold absorption bands).

**Table 2:** MIC of compounds **8a**, **8b**, **8d**, **8e**, **8f**, and **9** (stock solutions of the substances of approx. 10 mm in a volume of 1 mL DMSO (spectroscopy grade)).

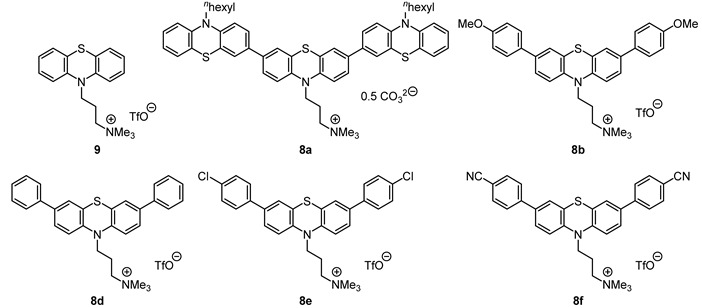									
				Antibacterial Activity MIC (µm)					
Compound	MW [g/mol]	Weighed Portion [mg]	Concentration [mm]	*M. tuberculosis*	*S. aureus* ATCC 29213	*E. coli* ATCC 25922	*P. aeruginosa* ATCC 27853	*A. baumannii* ATCC 747	*K. pneumoniae* ATCC 13883
**9**	448.5	4.53	10.1	100	>100	>100	>100	>100	>100
**8a**	1784.6	9.89	5.5	>100	>100	>100	>100	>100	>100
**8b**	660.8	6.62	10.0	25	12.5	>100	>100	100	>100
**8d**	600.7	6.01	10.0	12.5	6.25	25	100	25	12.5
**8e**	669.6	6.69	10.0	12.5	12.5	>100	>100	50	>100
**8f**	650.7	6.50	10.0	25	12.5	>100	>100	>100	>100

**Table 3:** Experimental details of the Suzuki synthesis of Boc-protected 3,7-di(hetero)aryl-substituted 10-(3-aminopropyl)10*H*-phenothiazines **7**.

Entry	Dibromide 5	Boronic Acid/Pinacol Boronate 6	CsF	Pd(PPh_3_)_4_	1,4-Dioxane	Compound 7 (Yield)
1	1.03 g (2.00 mmol)	1.81 g (4.40 mmol) of **6a**	1.94 g (12.8 mmol)	139 mg (0.12 mmol)	20 mL	1.56 g (85%) of **7a**
2	722 mg (1.40 mmol)	511 mg (3.36 mmol) of 4-methoxyphenyl boronic acid (**6b**)	1.36 g (8.97 mmol)	84 mg (0.07 mmol)	14 mL	630 mg (79%) of **7b**
3	514 mg (1.00 mmol)	462 mg (2.20 mmol) of pinacol 2-thienyl boronate (**6c**)	973 mg (6.40 mmol)	69 mg (0.06 mmol)	10 mL	510 mg (69%) of **7c**
4	771 mg (1.50 mmol)	402 mg (3.30 mmol) of phenyl boronic acid (**6d**)	1.45 g (9.60 mmol)	104 mg (0.09 mmol)	15 mL	560 mg (74%) of **7d**
5	771 mg (1.50 mmol)	787 mg (3.30 mmol) of 4-chlorophenyl boronic acid (**6e**)	1.46 g (9.60 mmol)	104 mg (0.09 mmol)	15 mL	660 mg (76%) of **7e**
6	722 mg (1.40 mmol)	1.36 g (8.97 mmol) of 4-cyanophenyl boronic acid (**6f**)	1.36 g (8.97 mmol)	84 mg (0.07 mmol)	14 mL	530 mg (67%) of **7f**

**Table 4:** Experimental details of the formation of 3,7-di(hetero)aryl-substituted 10-(3-trimethylammoniumpropyl)10*H*-phenothiazine salts **8**.

Entry	Compound 7	Dichloromethane	Trifluoro Acidic Acid	Potassium Carbonate	Methyl Iodide/Methyl Triflate	Compound 8 (Yield)
1	910 mg (0.99 mmol) of **7a**	10 mL	903 μL (7.92 mmol)	2.76 g (20.0 mmol)	623 μL (9.90 mmol) of methyl iodide	823 mg (93%) of **8a** ^1^
2	329 mg (0.58 mmol) of **7b**	6 mL	354 μL (4.63 mmol)	1.60 g (11.6 mmol)	634 μL (5.79 mmol) of methyl triflate	256 mg (67%) of **8b** ^2^
3	200 mg (0.39 mmol) of **7c**	4 mL	237 μL (3.08 mmol)	1.06 g (7.69 mmol)	420 μL (3.85 mmol) of methyl triflate	239 mg (59%) of **8c** ^2^
4	250 mg (0.49 mmol) of **7d**	5 mL	302 μL (3.93 mmol)	1.36 g (9.82 mmol)	537 μL (4.91 mmol) of methyl triflate	239 mg (81%) of **8d** ^2^
5	250 mg (0.43 mmol) of **7e**	4 mL	267 μL (3.49 mmol)	1.19 g (8.64 mmol)	473 μL (4.32 mmol) of methyl triflate	205 mg (71%) of **8e** ^2^
6	200 mg (0.44 mmol) of **7f**	4 mL	267 μL (3.49 mmol)	1.36 g (9.82 mmol)	493 μL (4.36 mmol) of methyl triflate	150 mg (53%) of **8f** ^2^

^1^ Obtained as carbonate. ^2^ Obtained as a triflate.

## Data Availability

All the data in this work are included in the manuscript and the Supplementary Materials.

## Supplementary Materials

The following supporting information can be downloaded at: https://www.mdpi.com/article/10.3390/molecules29092126/s1, ^1^H and ^13^C NMR spectra of compounds **7** and **8** (Figures S1–S20), correlation of *E*_0_^0/+1^ of compounds **7** and **8** against Hammett parameters (Tables S1–S4; Figures S21–S30).
