# Supplementary material for: Synthesis, Electronic, and Antibacterial Properties of 3,7-Di(hetero)aryl-substituted Phenothiazinyl N-Propyl Trimethylammonium Salts
Source: Molecules. 2024 May 3;29(9):2126. doi: 10.3390/molecules29092126 (PMC11085201; doi:10.3390/molecules29092126)
Supplement: Supplementary file 1 [file molecules-29-02126-s001.zip › molecules-2995884-supplementary.pdf]

Synthesis, electronic, and antibacterial properties of 3,7-di(hetero)arylsubstituted phenothiazinyl *N*-propyl trimethylammonium salts

Hilla Khelwati,[a] NN,[a] NN,[b] Rainer Kalscheuer,[b] Thomas J. J. Müller*[a]

[a] Heinrich Heine University Düsseldorf, Faculty of Mathematics and Natural Sciences, Institute of Organic Chemistry and Macromolecular Chemistry, Universitätsstrasse 1, D-40225 Düsseldorf, Germany

[b] Heinrich Heine University Düsseldorf, Faculty of Mathematics and Natural Sciences, Institute of Pharmaceutical Biology and Biotechnology, Universitätsstrasse 1, D-40225 Düsseldorf, Germany

E-mail: ThomasJJ.Mueller@hhu.de

Content

1. ^{1}H and ^{13}C NMR spectra of compounds 7 and 8 2

2. Correlation studies 12

2.1. Correlation of $E_0^{0/+1}$ of compounds 7 and 8 against Hammett parameters 12

2.2. Correlation of absorption, emission, and Stokes shift of compounds 7 and 8 against Hammett parameters 16

3. Antibacterial activity .... Fehler! Textmarke nicht definiert.

1. ^{1}H and ^{13}C NMR spectra of compounds 7 and 8

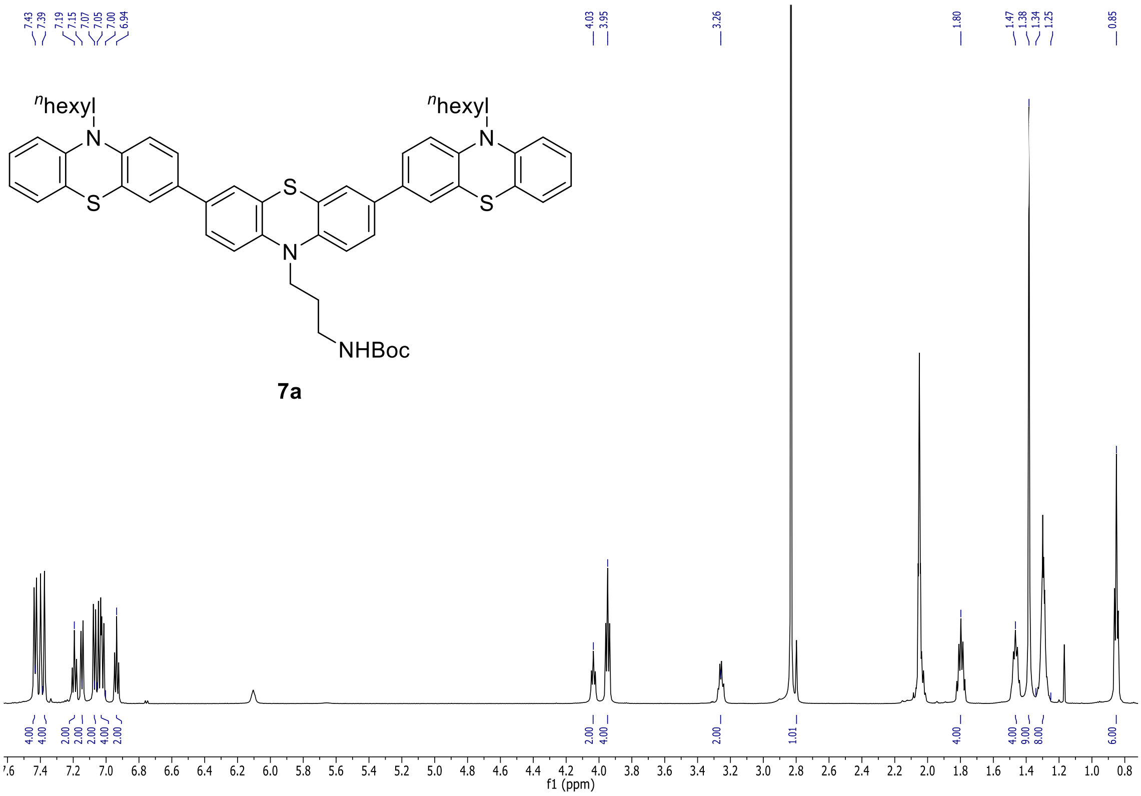

Figure S1. ^{1}H NMR spectrum of compound **7a** (600 MHz, acetone-d_6, 298 K).

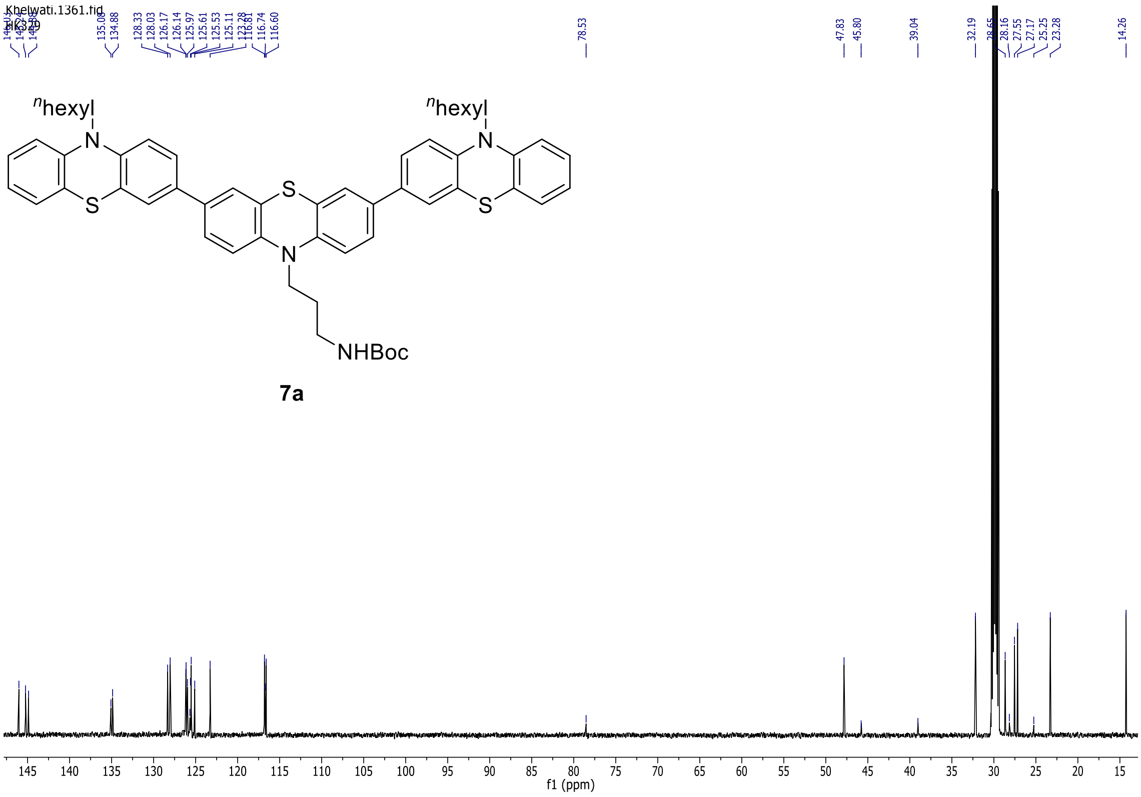

Figure S2. ^{13}C NMR spectrum of compound **7a** (151 MHz, acetone-d_6, 298 K).

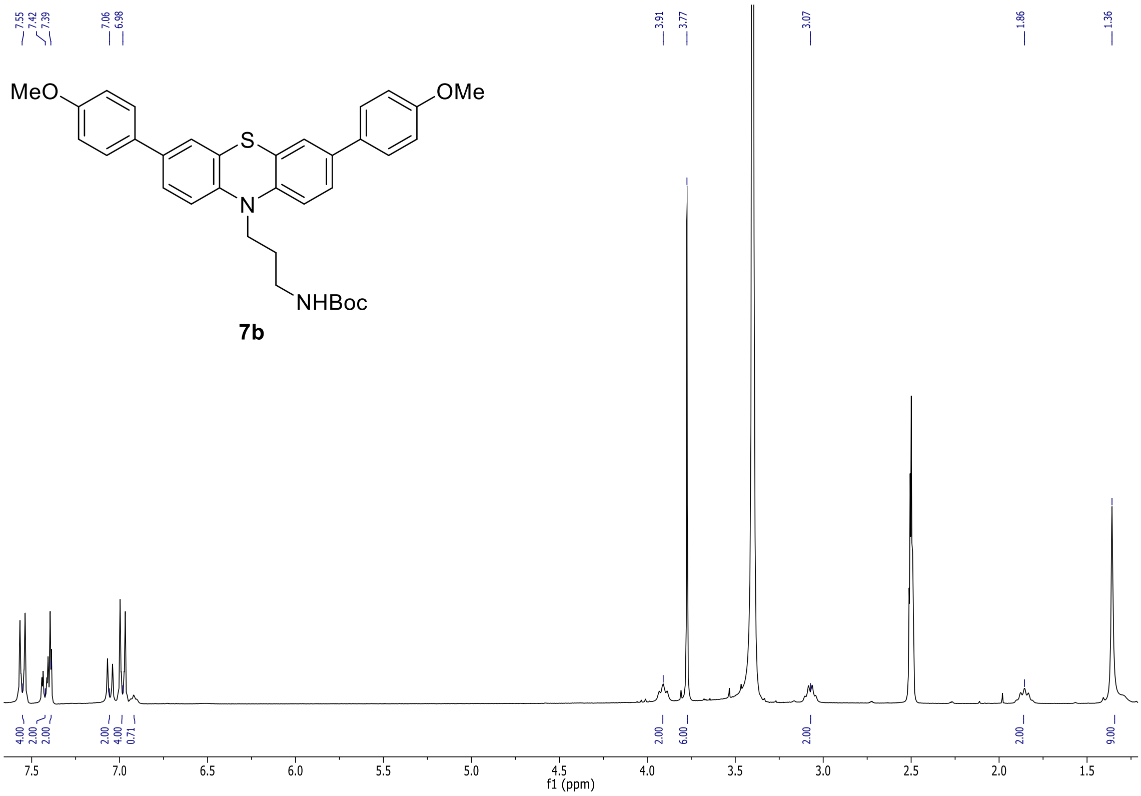

Figure S3. ^{1}H NMR spectrum of compound **7b** (300 MHz, DMSO-d_6, 298 K).

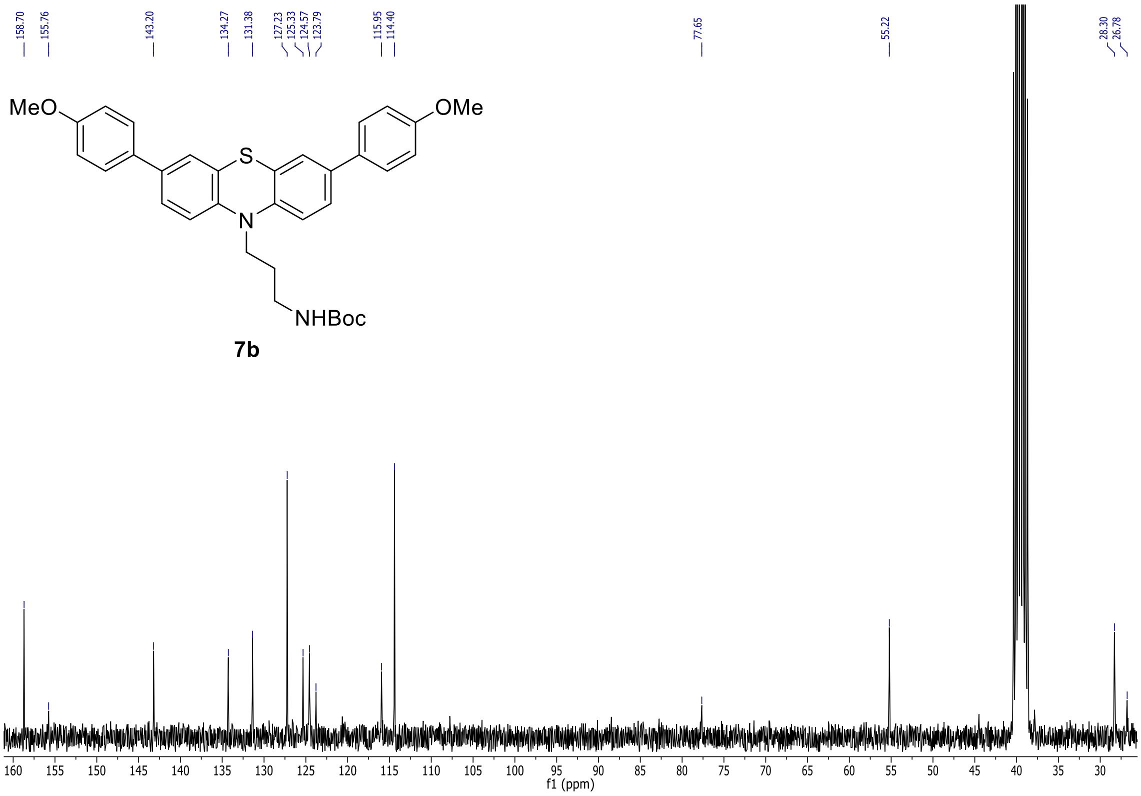

Figure S4. ^{13}C NMR spectrum of compound **7b** (75 MHz, DMSO-d_6, 298 K).

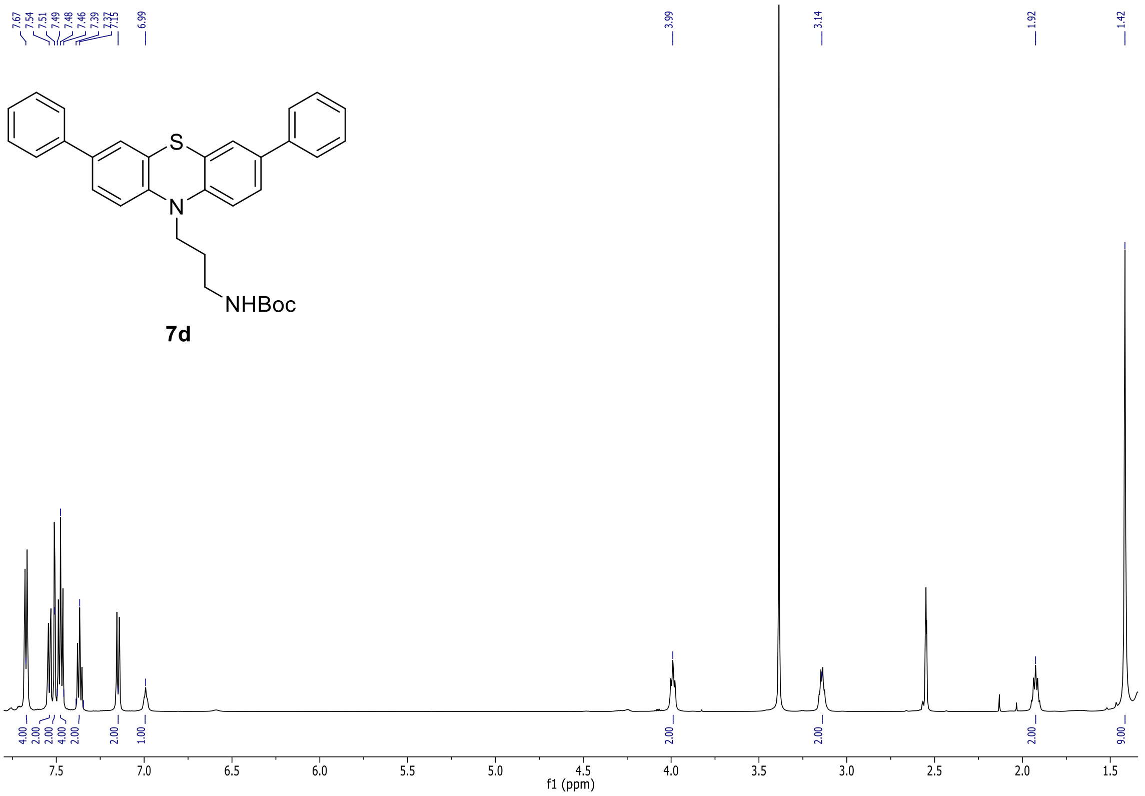

Figure S5. ^{1}H NMR spectrum of compound **7d** (600 MHz, acetone-d_6, 298 K).

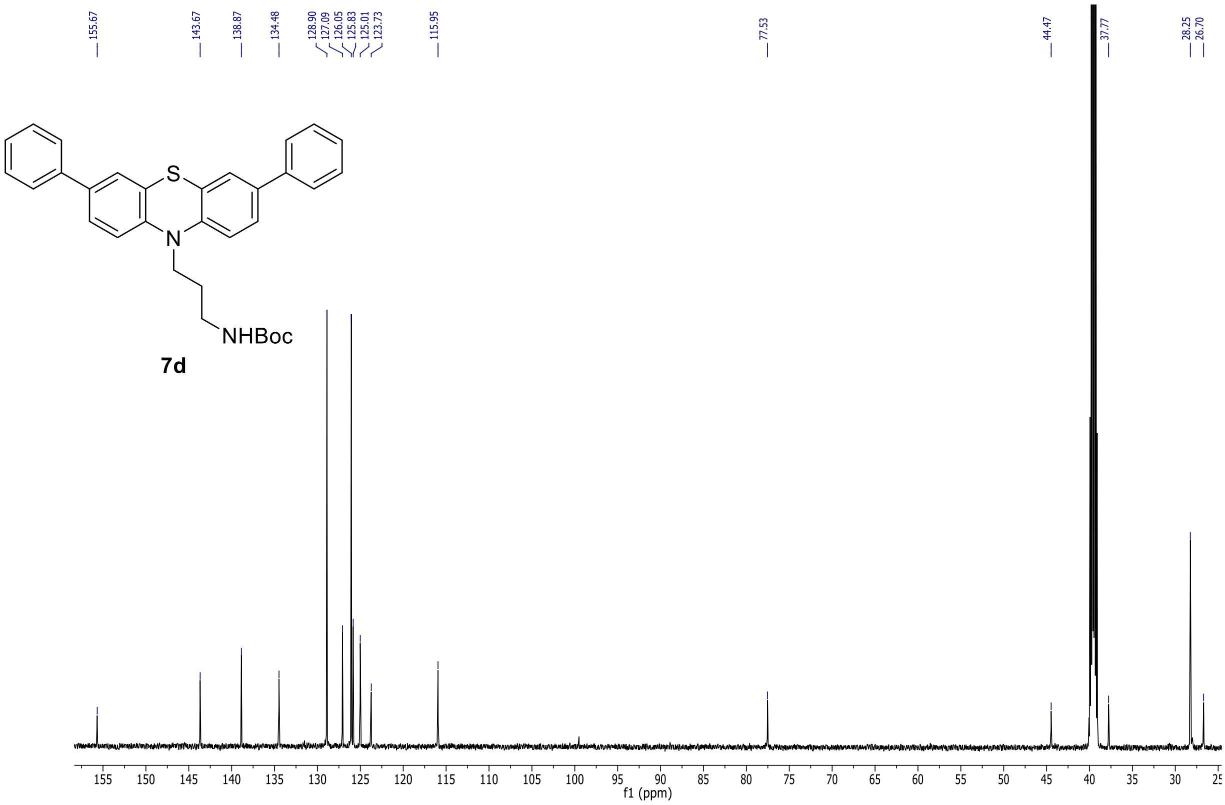

Figure S6. ^{13}C NMR spectrum of compound **7d** (151 MHz, acetone-d_6, 298 K).

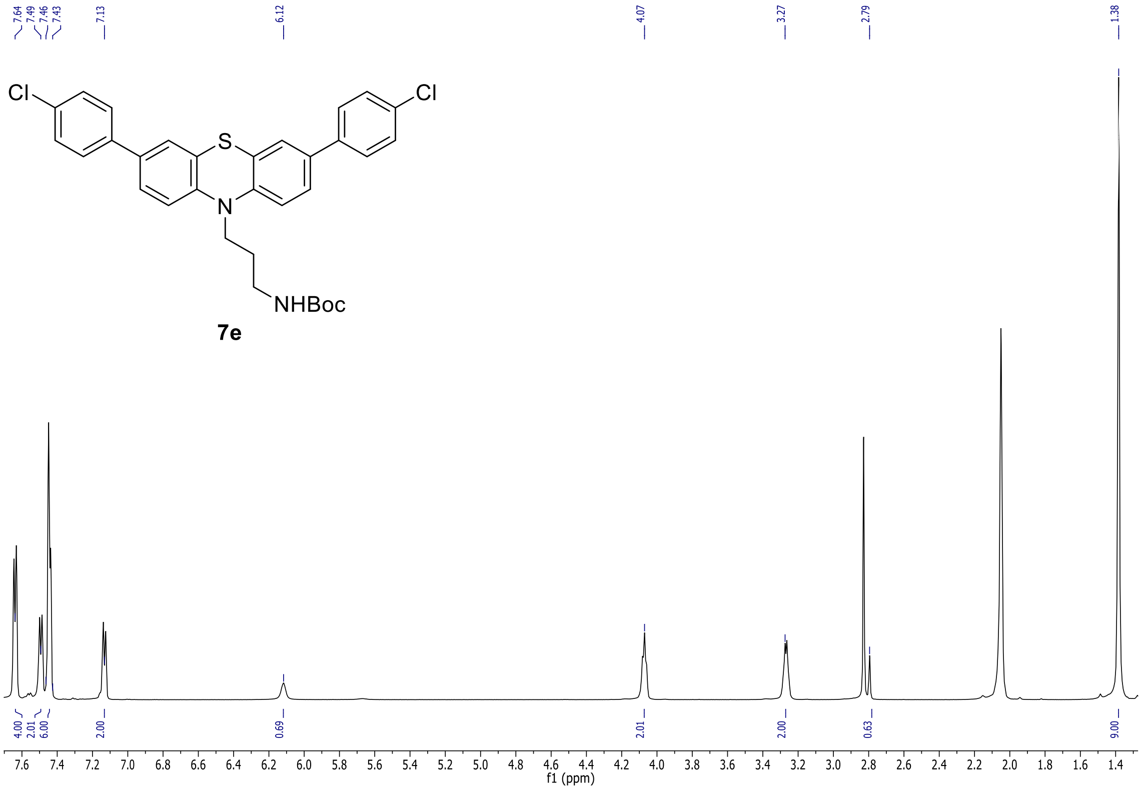

Figure S7. ^{1}H NMR spectrum of compound **7e** (600 MHz, acetone-d_6, 298 K).

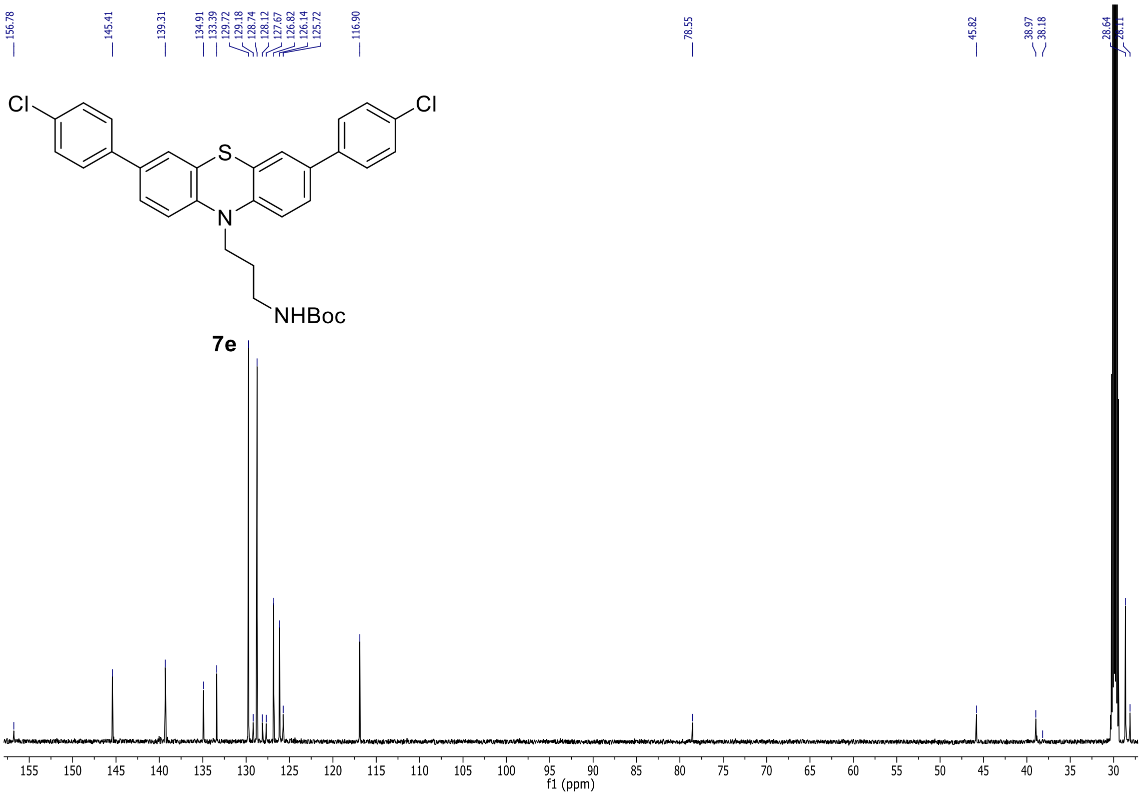

Figure S8. ^{13}C NMR spectrum of compound **7e** (151 MHz, acetone-d_6, 298 K).

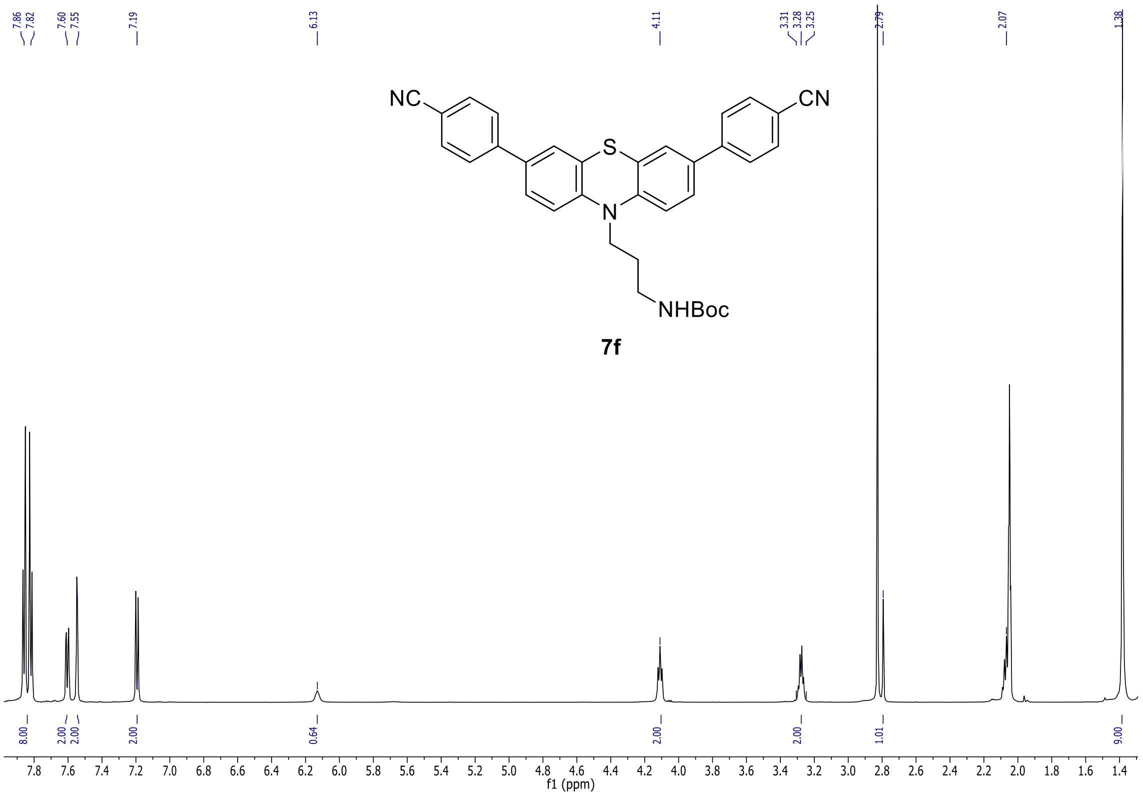

Figure S9. ^{1}H NMR spectrum of compound **7f** (600 MHz, acetone-d_6, 298 K).

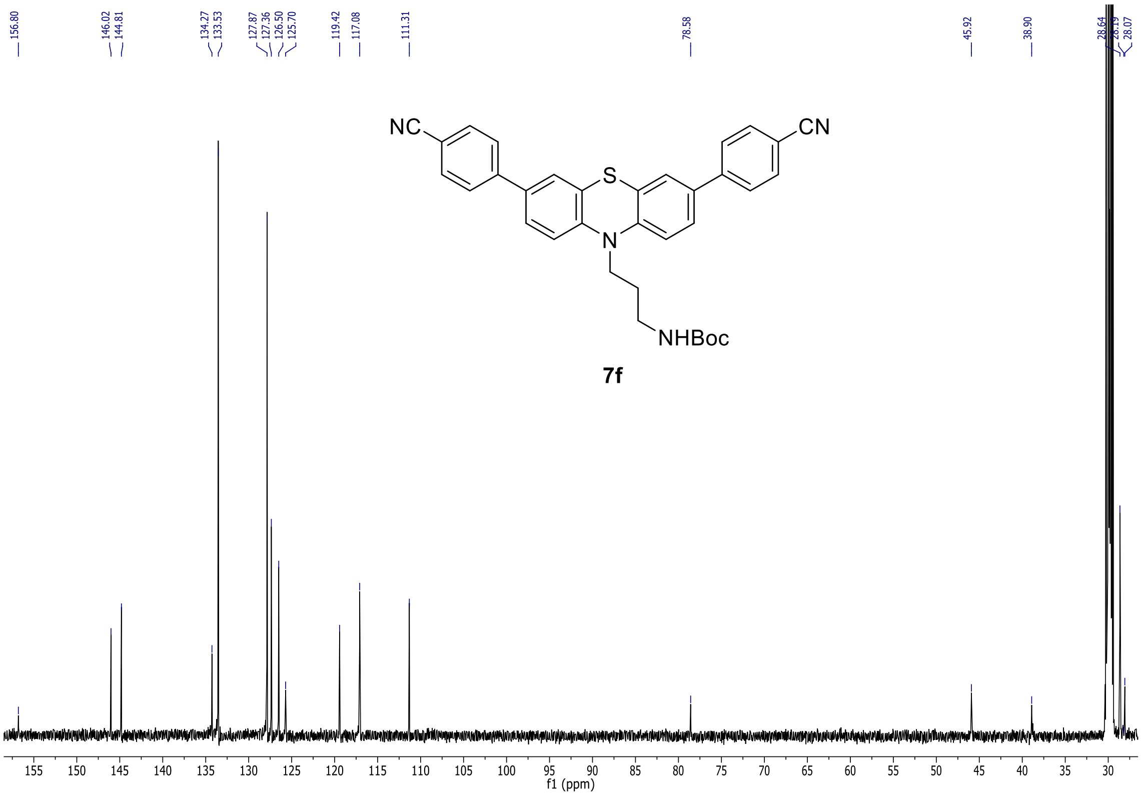

Figure S10. ^{13}C NMR spectrum of compound **7f** (151 MHz, acetone-d_6, 298 K).

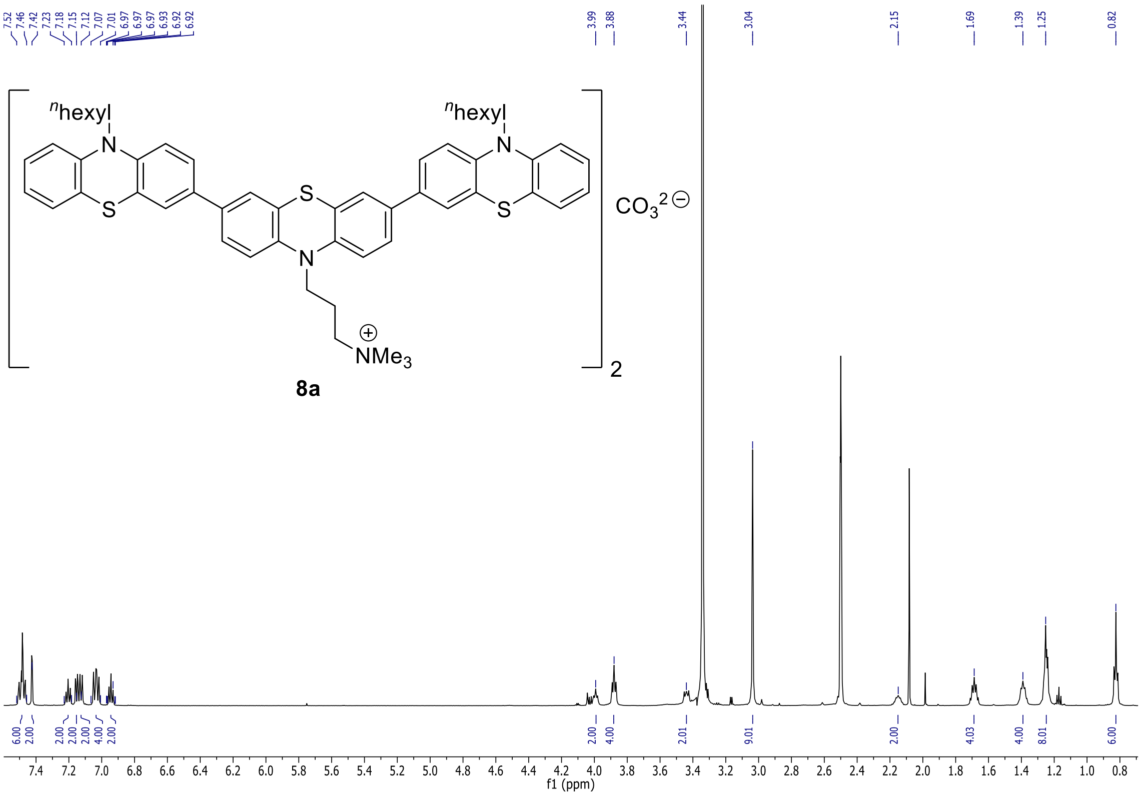

Figure S11. ^{1}H NMR spectrum of compound **8a** (600 MHz, DMSO-d_6, 298 K).

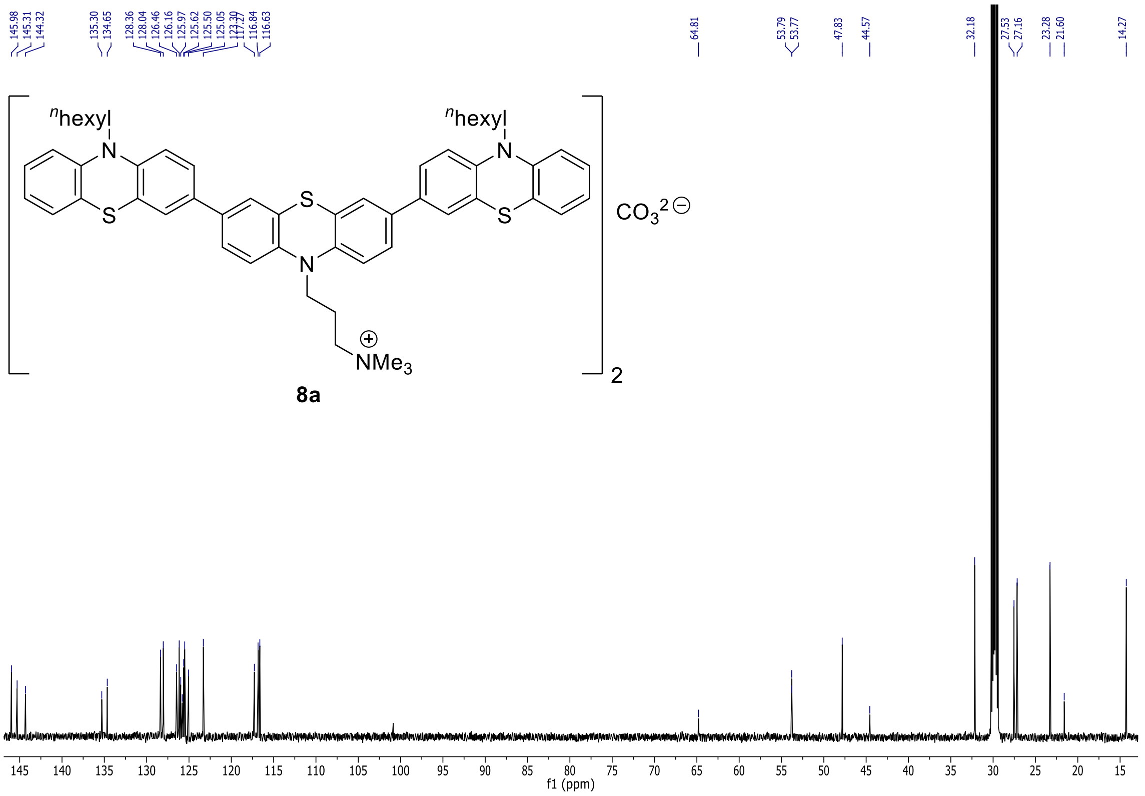

Figure S12. ^{13}C NMR spectrum of compound **8a** (151 MHz, acetone-d_6, 298 K).

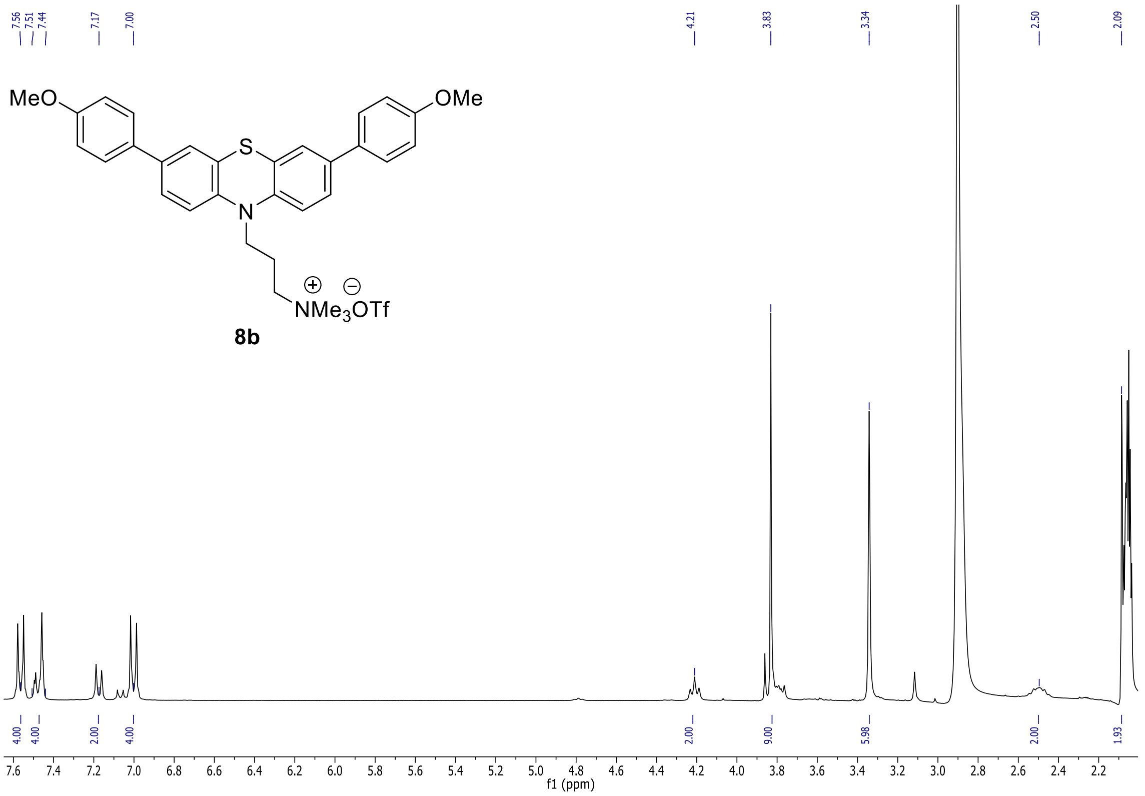

Figure S13. ^{1}H NMR spectrum of compound **8b** (300 MHz, acetone-d_6, 298 K).

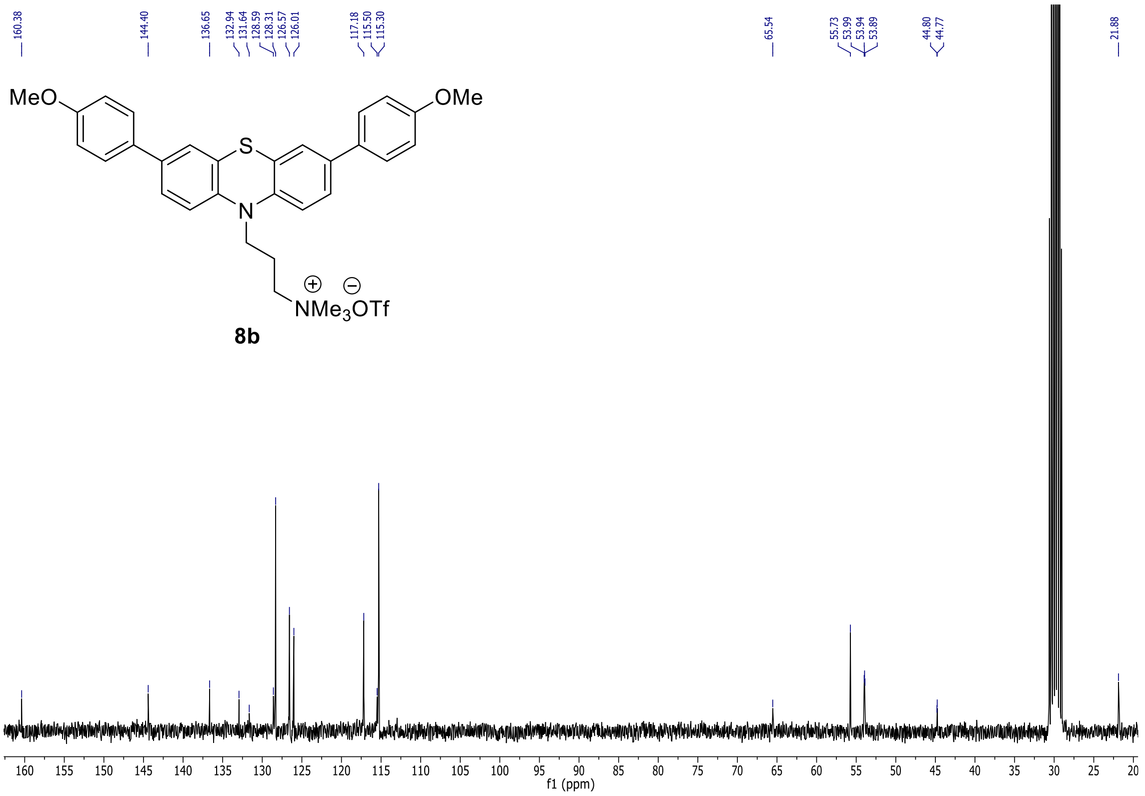

Figure S14. ^{13}C NMR spectrum of compound **8b** (75 MHz, acetone-d_6, 298 K).

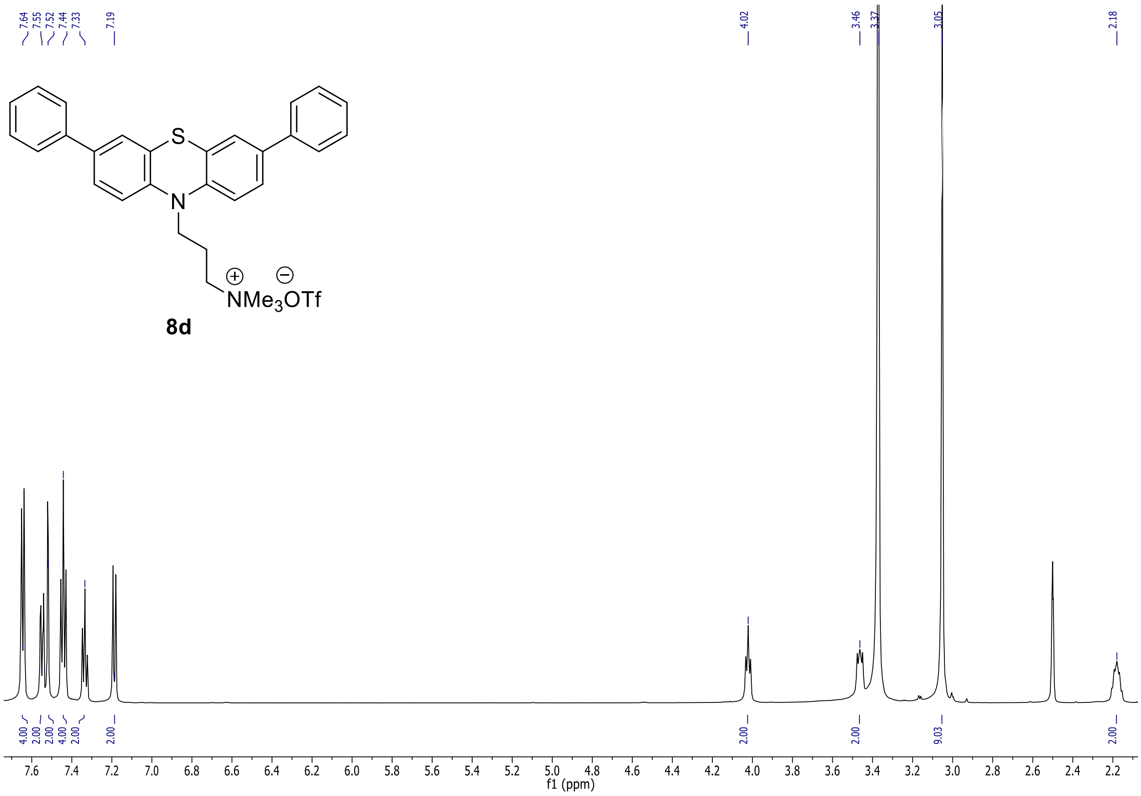

Figure S15. ^{1}H NMR spectrum of compound **8d** (600 MHz, DMSO-d_6, 298 K).

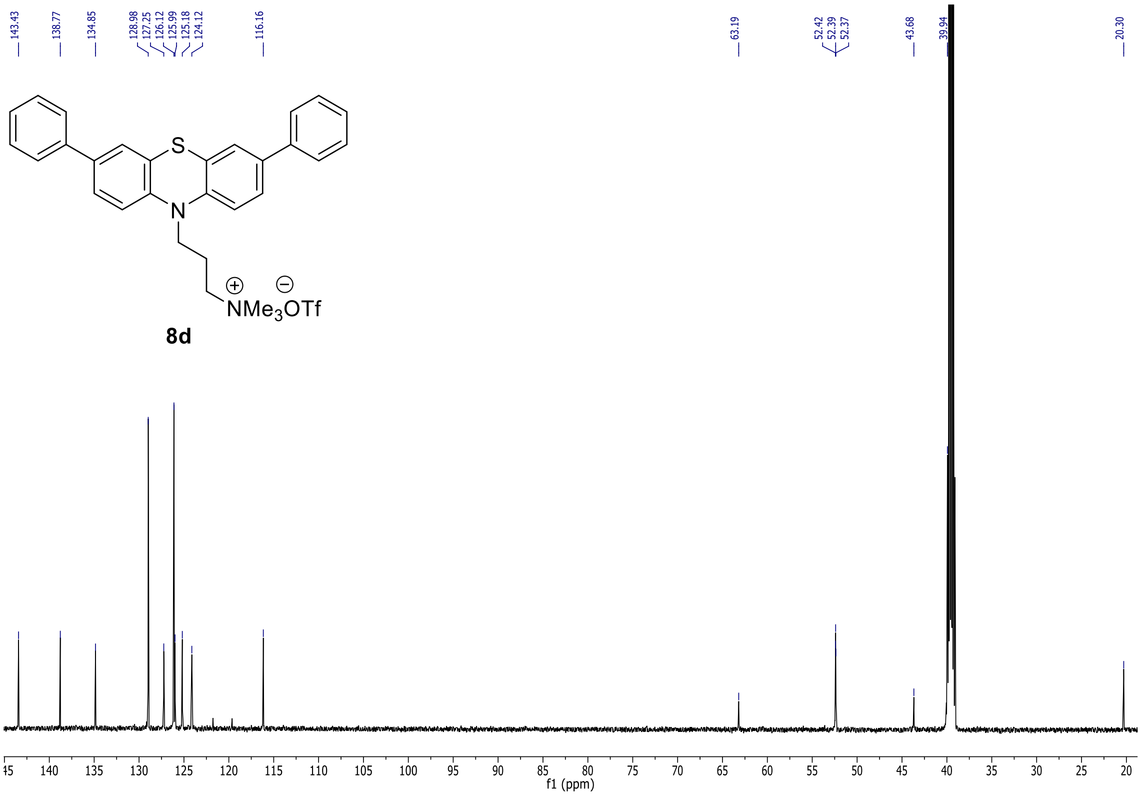

Figure S16. ^{13}C NMR spectrum of compound **8d** (151 MHz, DMSO-d_6, 298 K).

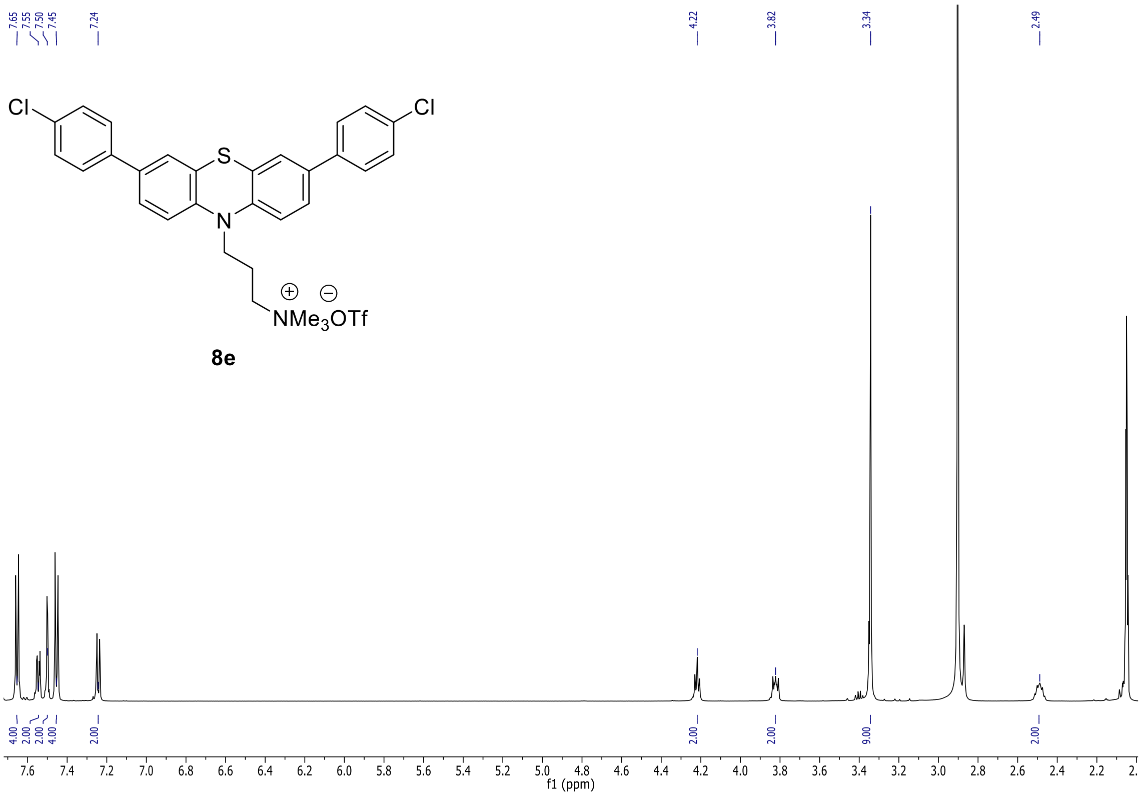

Figure S17. ^{1}H NMR spectrum of compound **8e** (600 MHz, acetone-d_6, 298 K).

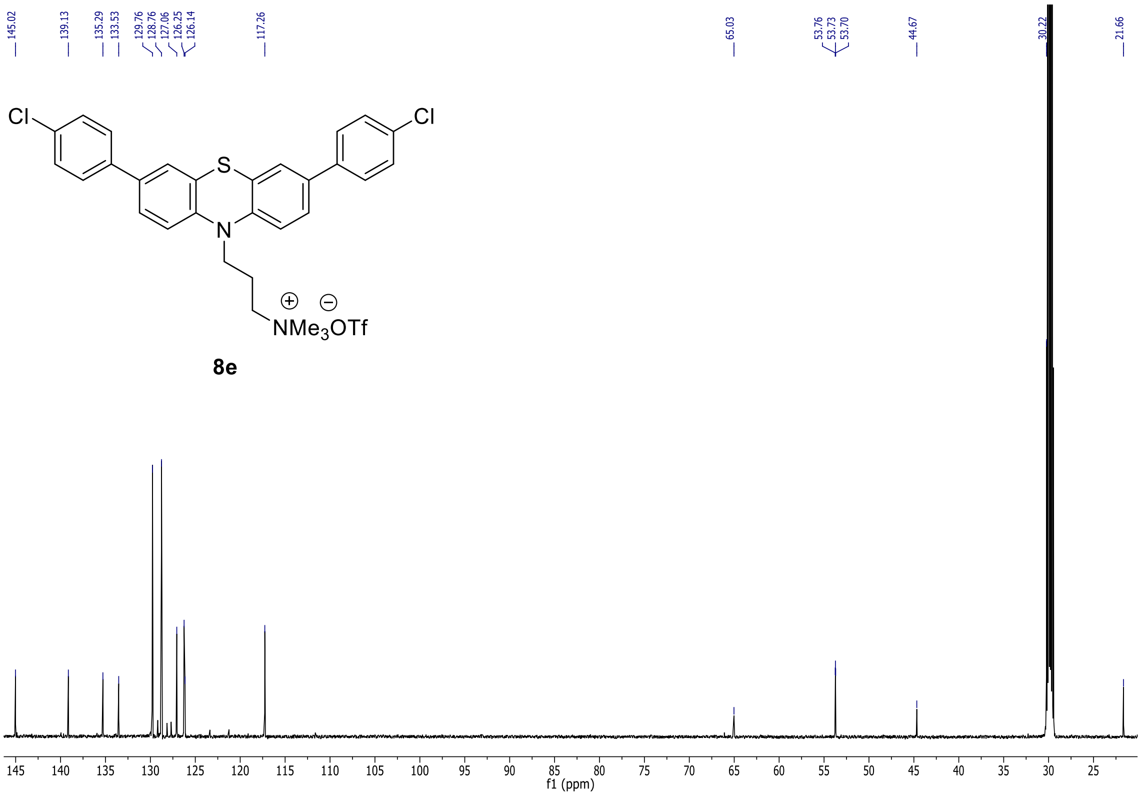

Figure S18. ^{13}C NMR spectrum of compound **8e** (151 MHz, acetone-d_6, 298 K).

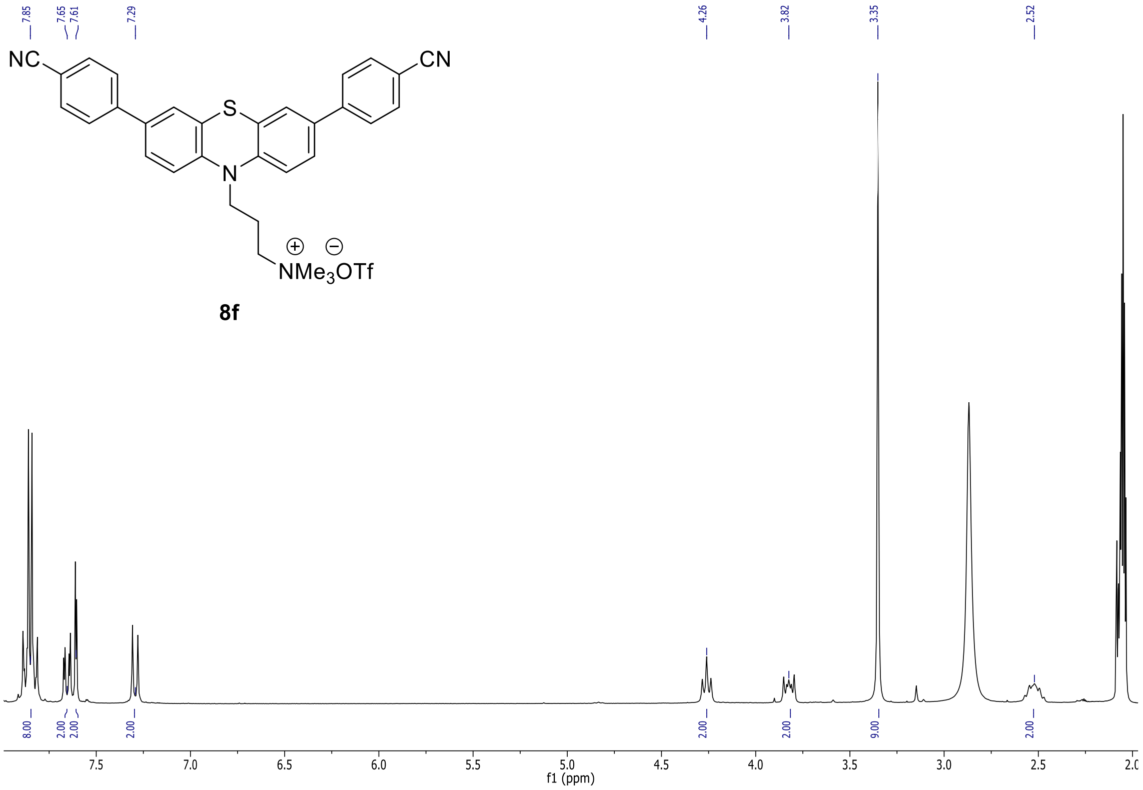

Figure S19. ^{1}H NMR spectrum of compound **8f** (300 MHz, acetone-d$_6$, 298 K).

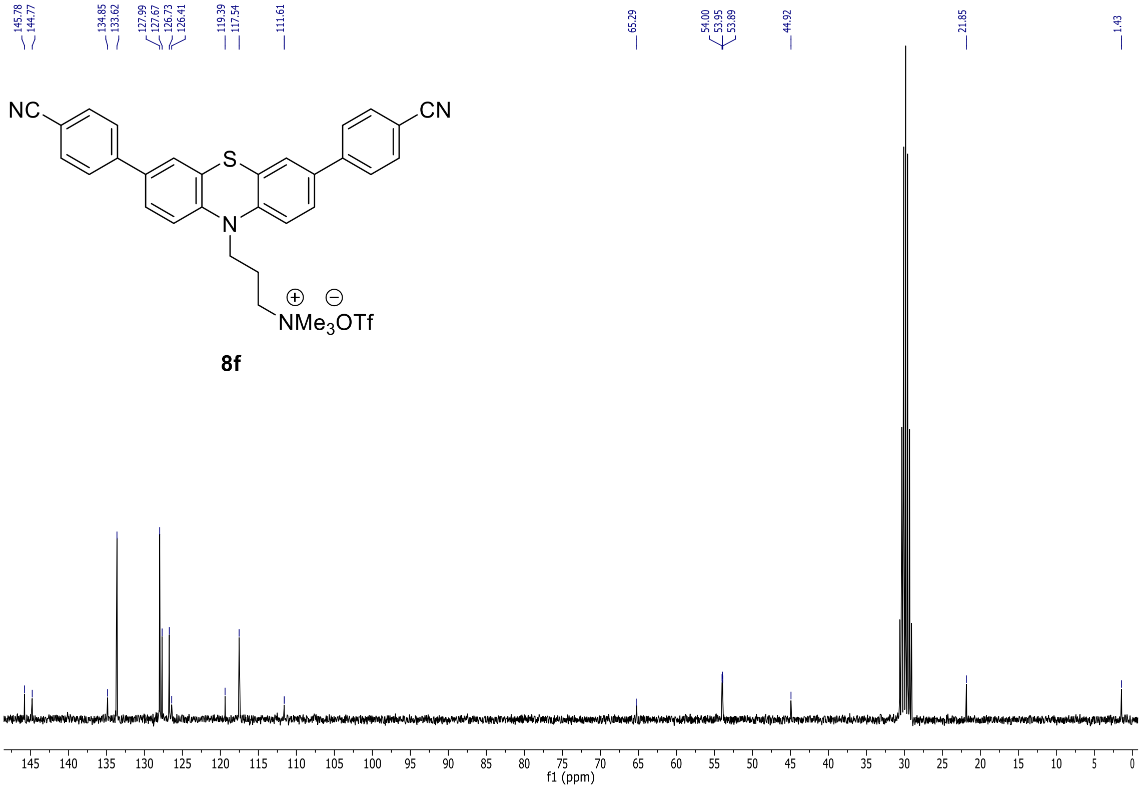

Figure S20. ^{13}C NMR spectrum of compound **8f** (75 MHz, acetone-d$_6$, 298 K).

2. Correlation studies

2.1. Correlation of $E_0^{0/+1}$ of compounds 7 and 8 against Hammett parameters

Compounds 7

Table S1. Selected Hammett parameters and oxidation potentials $E_0^{0/+1}$ of compounds **7b-f**.

compounds	σ_p	σ_{p+}	σ_{p-}	σ_R	σ_{R+}	$E_0^{0/+1}$ [V]
**7b**	-0.27	-0.78	-0.27	-0.43	-1.07	0.64
**7c**	0.05	-0.43	0.05	-0.14	-0.56	0.69
**7d**	0.06	-0.18	0.06	-0.08	-0.3	0.7
**7e**	0.23	0.11	0.23	-0.16	-0.31	0.73
**7f**	0.66	0.66	0.66	0.16	0.15	0.83

σ_p: $E_0^{0/+1} = 0.2067 \cdot \sigma_p + 0.6878$ [V] ($R^2 = 0.9903$)

σ_{p+}: $E_0^{0/+1} = 0.1265 \cdot \sigma_{p+} + 0.7337$ [V] ($R^2 = 0.9631$)

σ_{p-}: $E_0^{0/+1} = 0.1464 \cdot \sigma_{p-} + 0.6846$ [V] ($R^2 = 0.9491$)

σ_R: $E_0^{0/+1} = 0.3091 \cdot \sigma_R + 0.7582$ [V] ($R^2 = 0.8537$)

σ_{R+}: $E_0^{0/+1} = 0.1482 \cdot \sigma_{R+} + 0.78$ [V] ($R^2 = 0.877$)

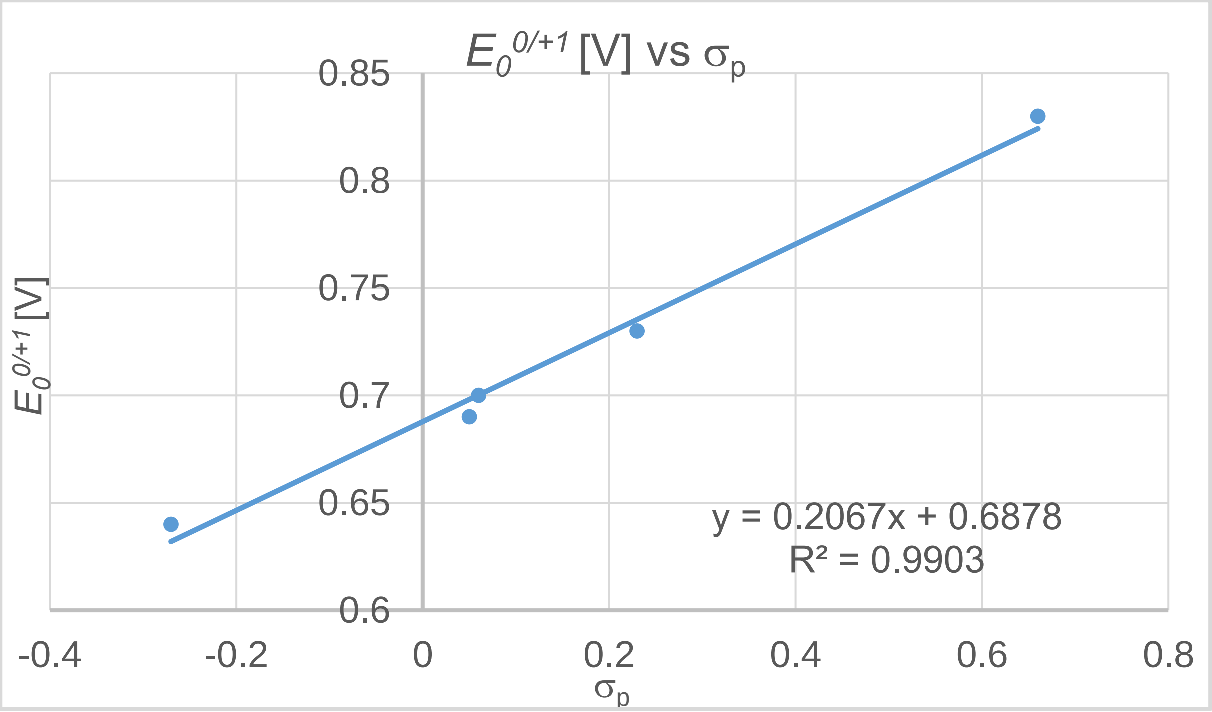

Figure S21. Hammett correlation of $E_0^{0/+1}$ of compounds **7b-f** against σ_p.

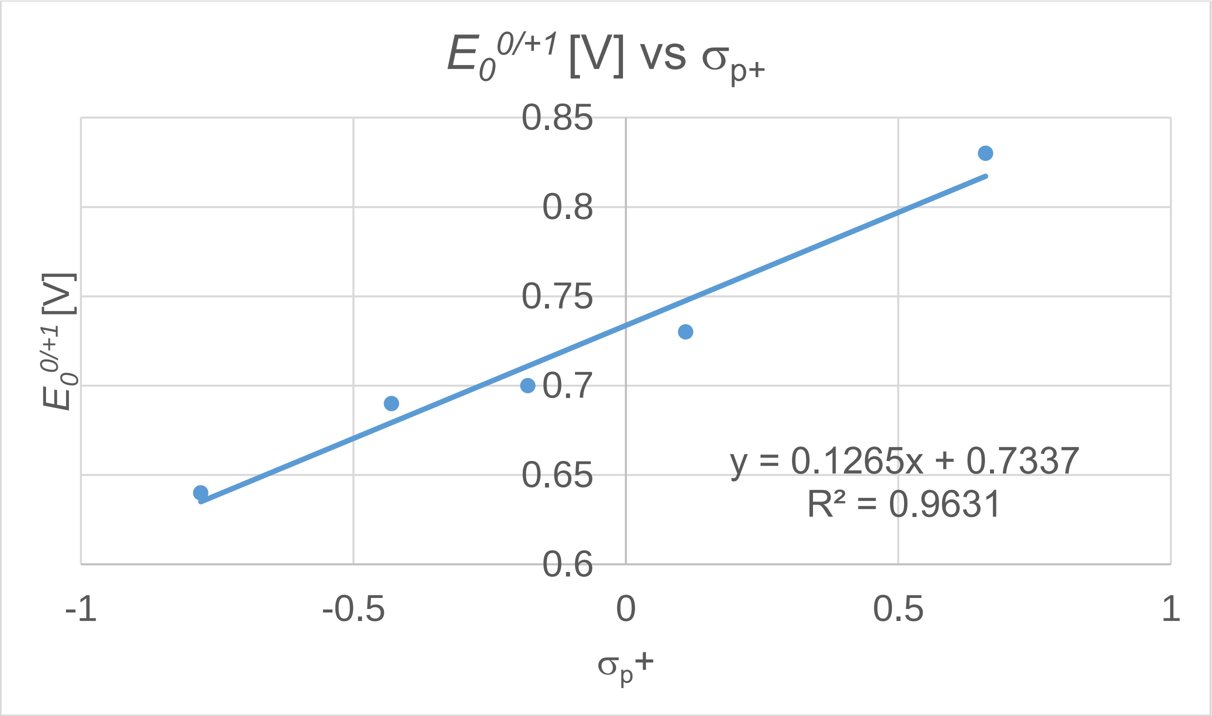

Figure S22. Hammett correlation of $E_0^{0/+1}$ of compounds **7b-f** against σ_{p+}.

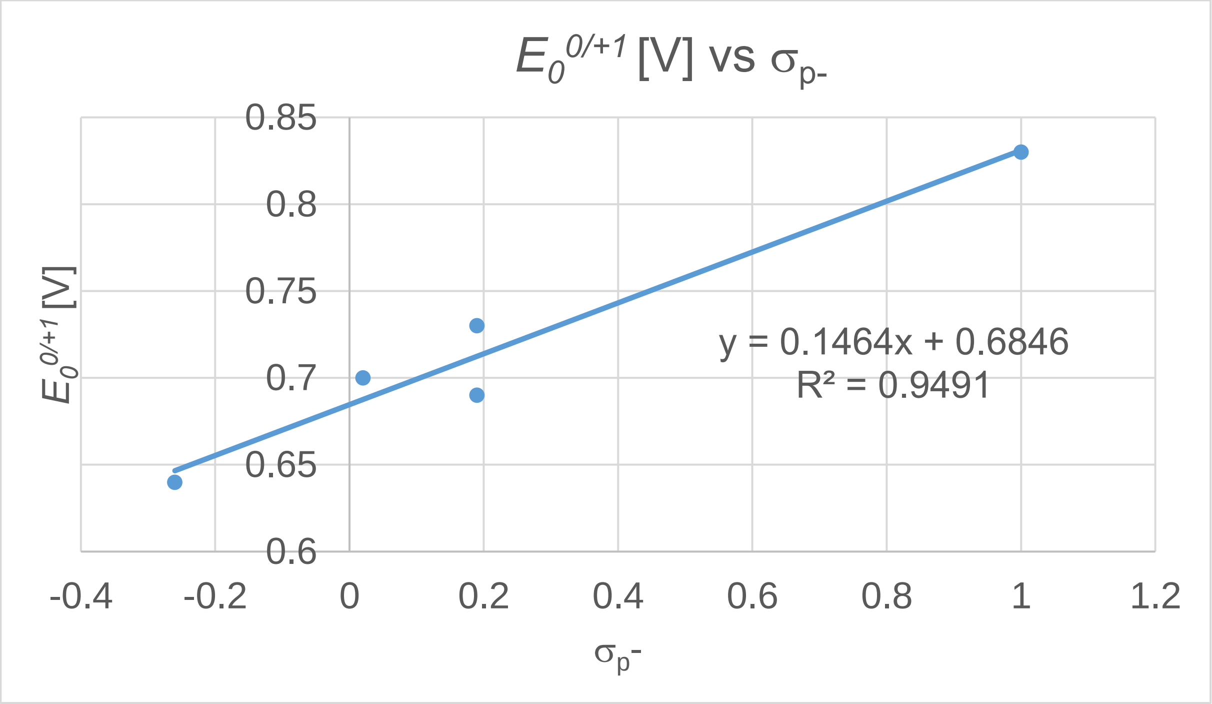

Figure S23. Hammett correlation of $E_0^{0/+1}$ of compounds **7b-f** against σ_{p-}.

Compounds 8

Table S2. Selected Hammett parameters and oxidation potentials $E_0^{0/+1}$ of compounds **8b-f**.

compounds	σ_p	σ_{p+}	σ_{p-}	σ_R	σ_{R+}	$E_0^{0/+1}$ [V]
**8b**	-0.27	-0.78	-0.27	-0.43	-1.07	0.68
**8c**	0.05	-0.43	0.05	-0.14	-0.56	0.72
**8d**	0.06	-0.18	0.06	-0.08	-0.3	0.73
**8e**	0.23	0.11	0.23	-0.16	-0.31	0.77
**8f**	0.66	0.66	0.66	0.16	0.15	0.84

σ_p: $E_0^{0/+1}$ = 0.1772·σ_p + 0.7221 [V] (R^2 = 0.9855)

σ_{p+}: $E_0^{0/+1}$ = 0.1099·σ_{p+} + 0.7616 [V] (R^2 = 0.9839)

σ_{p-}: $E_0^{0/+1}$ = 0.1229·σ_{p-} + 0.72 [V] (R^2 = 0.9056)

σ_R: $E_0^{0/+1}$ = 0.3091·σ_R + 0.7582 [V] (R^2 = 0.8537)

σ_{R+}: $E_0^{0/+1}$ = 0.2579·σ_{R+} + 0.7815 [V] (R^2 = 0.8046)

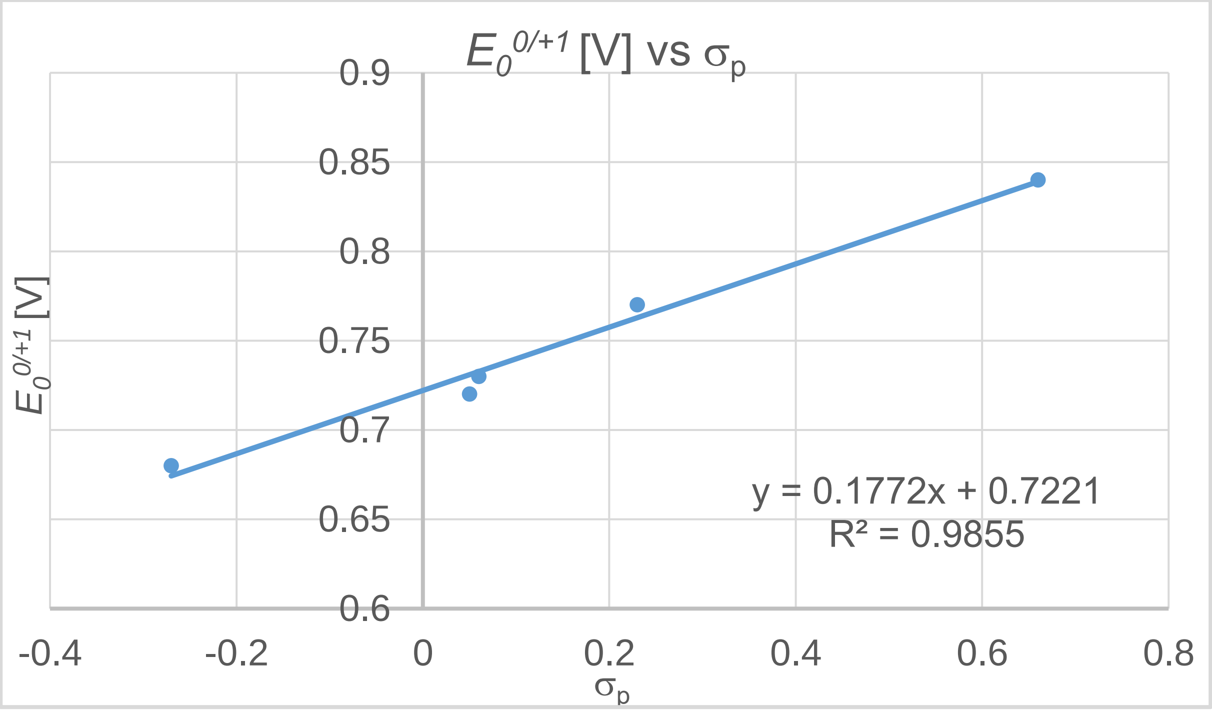

Figure S24. Hammett correlation of $E_0^{0/+1}$ of compounds **8b-f** against σ_p.

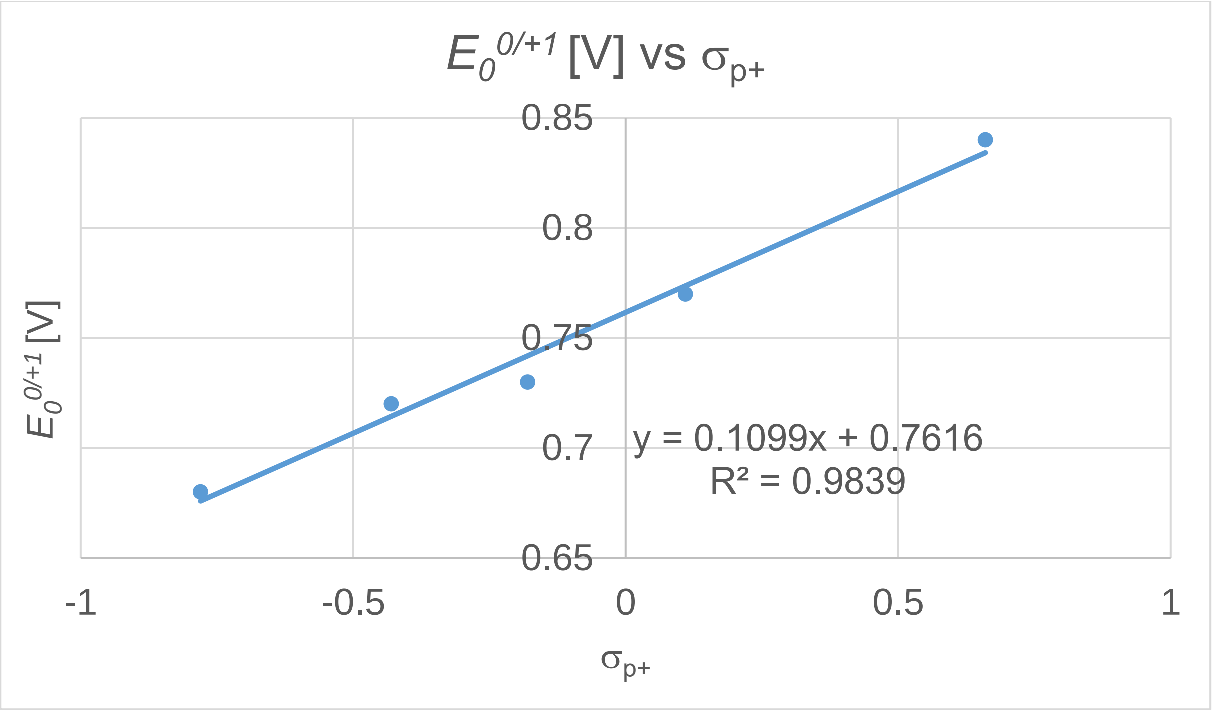

Figure S25. Hammett correlation of $E_0^{0/+1}$ of compounds **8b-f** against σ_{p+}.

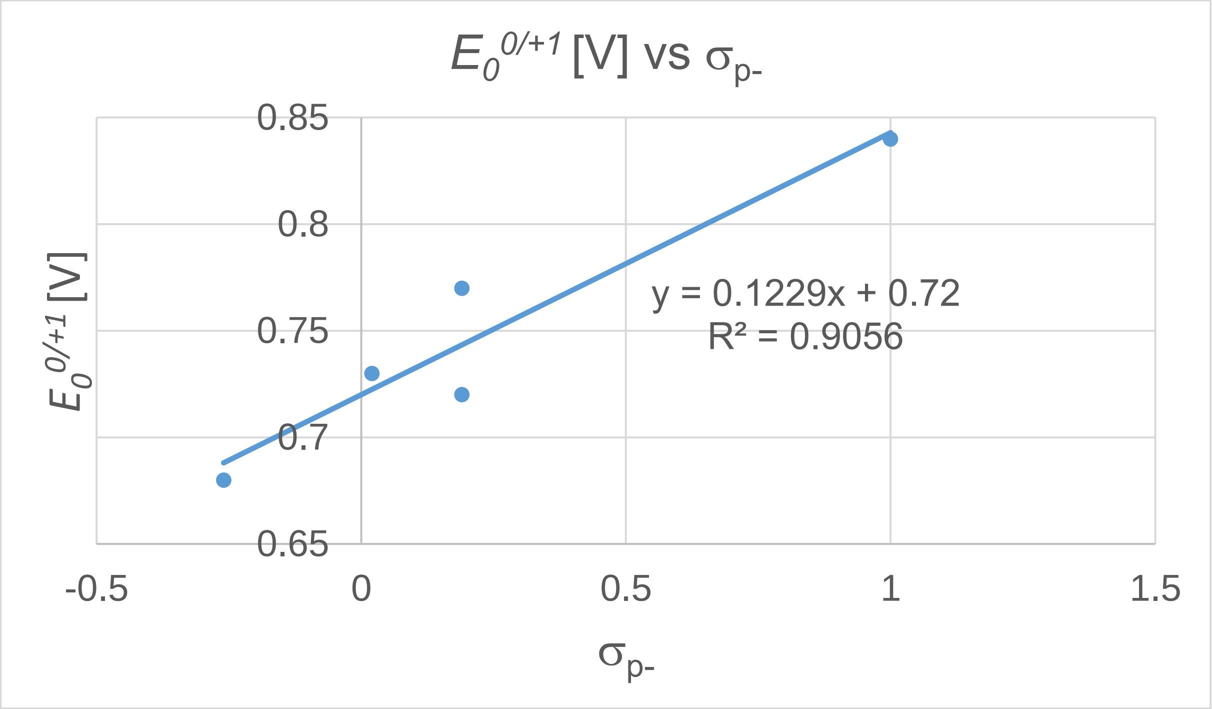

Figure S26. Hammett correlation of $E_0^{0/+1}$ of compounds **8b-f** against σ_{p-}.

2.2. Correlation of absorption, emission, and Stokes shift of compounds 7 and 8 against Hammett parameters

Compounds 7

Table S3. Selected Hammett parameters and $\lambda_{max,abs}$, $\tilde{\nu}_{max,abs}$, $\lambda_{max,em}$, $\tilde{\nu}_{max,em}$, and $\Delta\tilde{\nu}$ of compounds **7b-f**.

σ_p	σ_{p+}	σ_{p-}	σ_R	σ_{R+}	$\lambda_{max,abs}$ [nm]	$\tilde{\nu}_{max,abs}$ [cm^{-1}]	$\lambda_{max,em}$ [nm]	$\tilde{\nu}_{max,em}$ [cm^{-1}]	$\Delta\tilde{\nu}$ [cm^{-1}]
-0.27	-0.78	-0.27	-0.43	-1.07	330	30300	461	21700	8600
0.05	-0.43	0.05	-0.14	-0.56	347	28800	488	20500	8300
0.06	-0.18	0.06	-0.08	-0.3	330	30300	471	21200	9100
0.23	0.11	0.23	-0.16	-0.31	333	30000	488	20500	9500
0.66	0.66	0.66	0.16	0.15	373	26800	520	19200	7600

Absorption bands

σ_p: $\tilde{\nu}_{max,abs} = -3636.2 \cdot \sigma_p + 29784$ [cm^{-1}] ($R^2 = 0.6796$)

σ_{p+}: $\tilde{\nu}_{max,abs} = -1962.7 \cdot \sigma_{p+} + 29009$ [cm^{-1}] ($R^2 = 0.514$)

σ_{p-}: $\tilde{\nu}_{max,abs} = -2987.8 \cdot \sigma_{p-} + 29934$ [cm^{-1}] ($R^2 = 0.8763$)

σ_R: $\tilde{\nu}_{max,abs} = -5574.6 \cdot \sigma_R + 28528$ [cm^{-1}] ($R^2 = 0.6154$)

σ_{R+}: $\tilde{\nu}_{max,abs} = -2272.2 \cdot \sigma_{R+} + 28303$ [cm^{-1}] ($R^2 = 0.4568$)

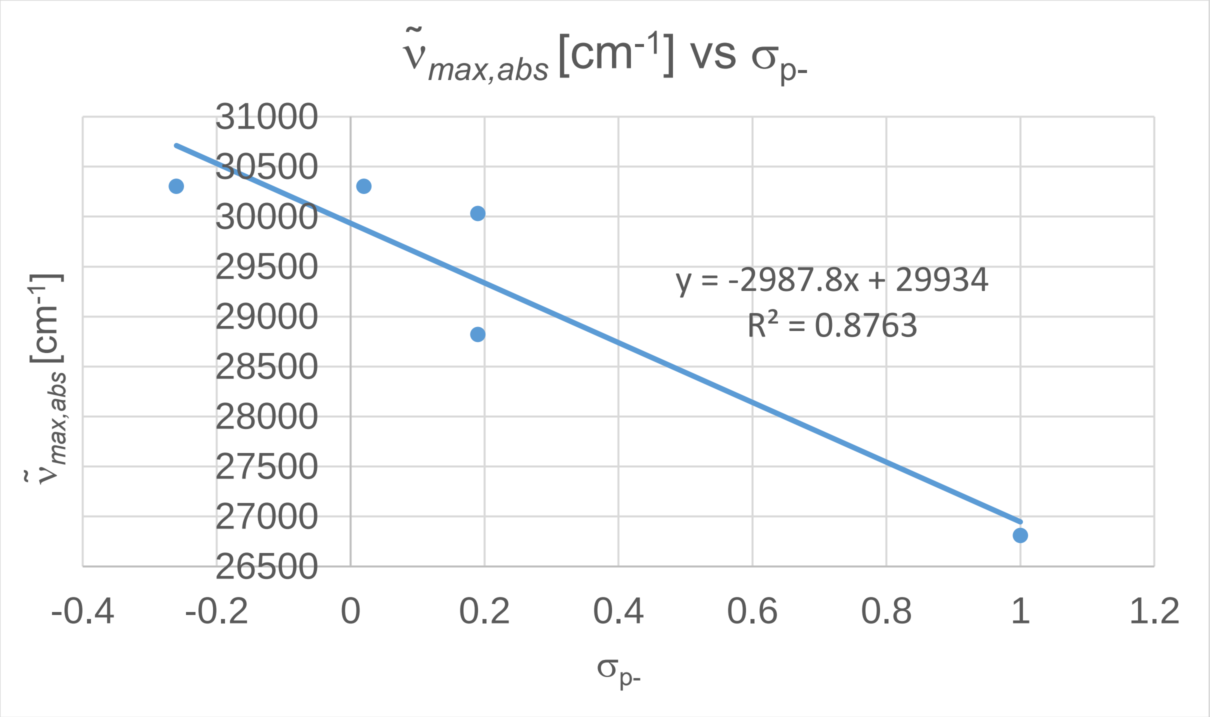

Figure S27. Hammett correlation of $E_0^{0/+1}$ of compounds **8b-f** against σ_{p-}.

Emission bands

σ_p: $\tilde{\nu}_{max,em}$ = -2627.5·σ_p + 21011 [cm^{-1}] (R^2 = 0.9129)

σ_{p+}: $\tilde{\nu}_{max,em}$ = -1518.9·σ_{p+} + 20439 [cm^{-1}] (R^2 = 0.792)

σ_{p-}: $\tilde{\nu}_{max,em}$ = -1946·σ_{p-} + 21071 [cm^{-1}] (R^2 = 0.9564)

σ_R: $\tilde{\nu}_{max,em}$ = -3878.2·σ_R + 20123 [cm^{-1}] (R^2 = 0.7664)

σ_{R+}: $\tilde{\nu}_{max,em}$ = -1778.9·σ_{R+} + 19884 [cm^{-1}] (R^2 = 0.7204)

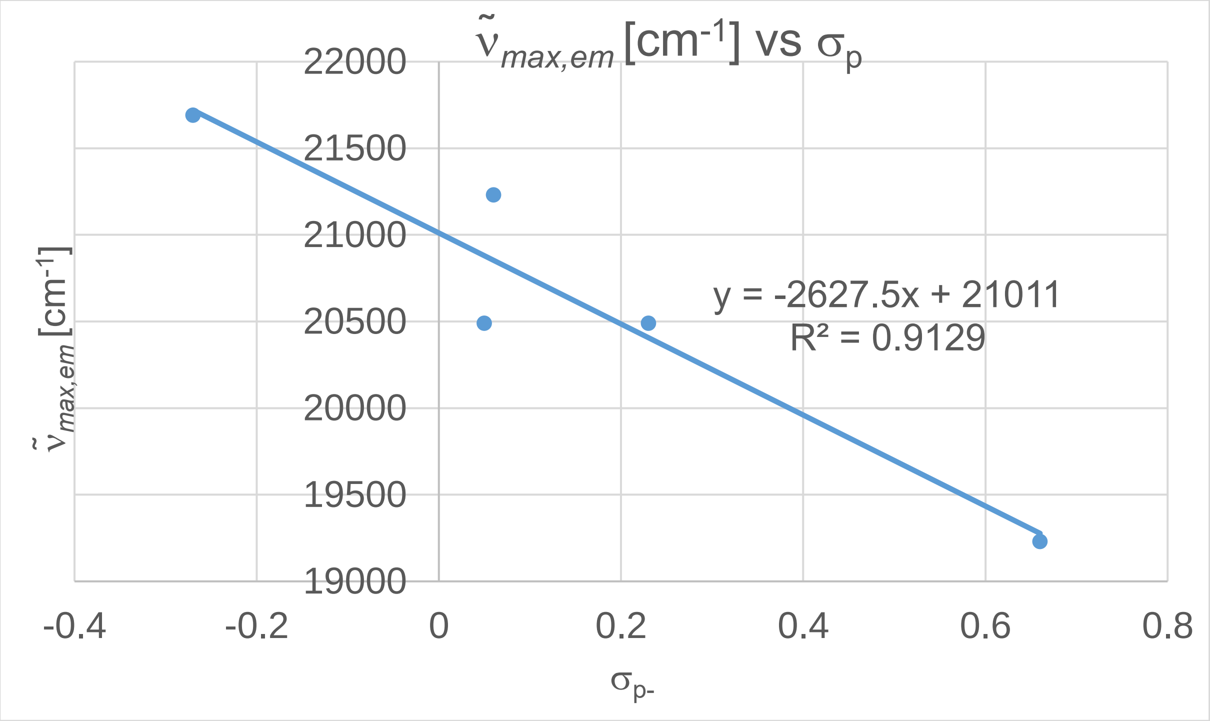

Figure S28. Hammett correlation of $E_0^{0/+1}$ of compounds **8b-f** against σ_p.

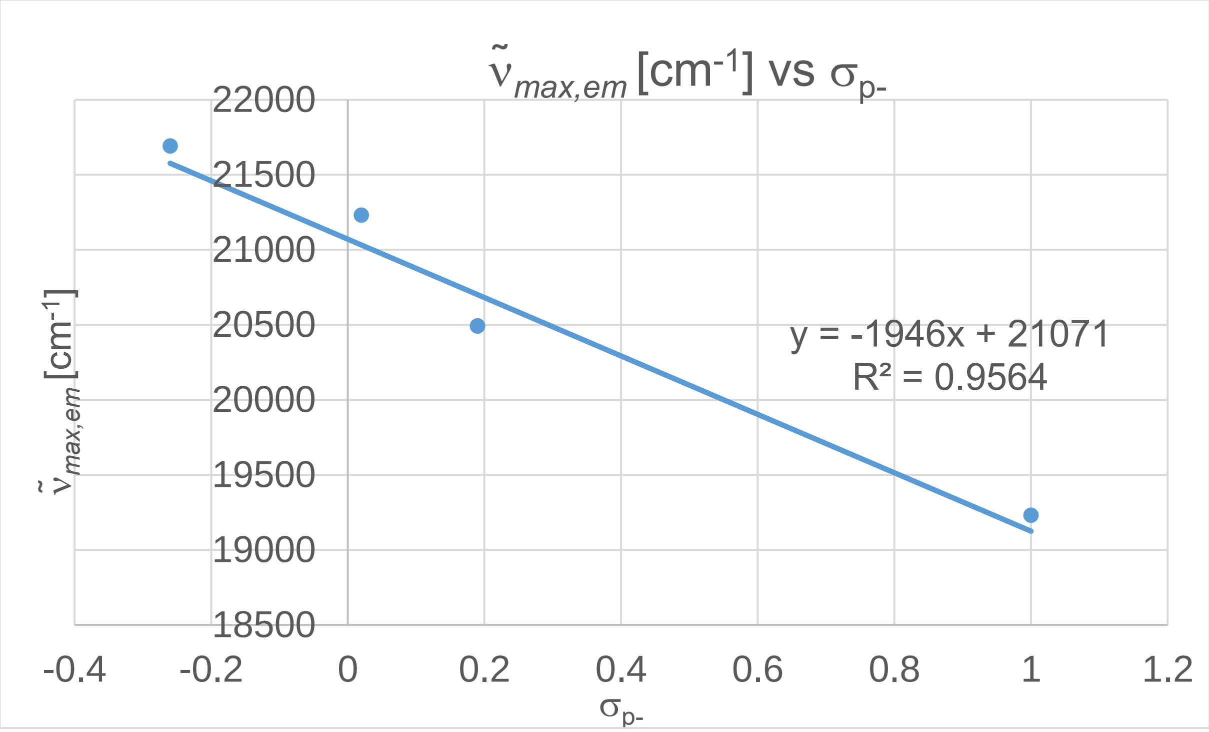

Figure S29. Hammett correlation of $E_0^{0/+1}$ of compounds **8b-f** against σ_{p-}.

Stokes shifts

σ_p: $\Delta\tilde{\nu} = -1008.8\cdot\sigma_p + 8772.6$ [cm^{-1}] ($R^2 = 0.2116$)

σ_{p+}: $\Delta\tilde{\nu} = -443.81\cdot\sigma_{p+} + 8570.3$ [cm^{-1}] ($R^2 = 0.1063$)

σ_{p-}: $\Delta\tilde{\nu} = -1041.8\cdot\sigma_{p-} + 8862.8$ [cm^{-1}] ($R^2 = 0.4309$)

σ_R: $\Delta\tilde{\nu} = -1696.4\cdot\sigma_R + 8404.8$ [cm^{-1}] ($R^2 = 0.2305$)

σ_{R+}: $\Delta\tilde{\nu} = -493.28\cdot\sigma_{R+} + 8419.1$ [cm^{-1}] ($R^2 = 0.0871$)

Compounds 8

Table S4. Selected Hammett parameters and $\lambda_{max,abs}$, $\tilde{\nu}_{max,abs}$, $\lambda_{max,em}$, $\tilde{\nu}_{max,em}$, and $\Delta\tilde{\nu}$ of compounds **8b-f**.

σ_p	σ_{p+}	σ_{p-}	σ_R	σ_{R+}	$\lambda_{max,abs}$ [nm]	$\tilde{\nu}_{max,abs}$ [cm^{-1}]	$\lambda_{max,em}$ [nm]	$\tilde{\nu}_{max,em}$ [cm^{-1}]	$\Delta\tilde{\nu}$ [cm^{-1}]
-0.27	-0.78	-0.27	-0.43	-1.07	324	30900	455	22000	8900
0.05	-0.43	0.05	-0.14	-0.56	326	30700	466	21500	9200
0.06	-0.18	0.06	-0.08	-0.3	323	31000	456	21900	9000
0.23	0.11	0.23	-0.16	-0.31	342	29200	478	20900	8300
0.66	0.66	0.66	0.16	0.15	359	27900	500	20000	7900

Absorption

σ_p: $\tilde{\nu}_{max,abs} = -3630 \cdot \sigma_p + 30449$ [cm^{-1}] ($R^2 = 0.8356$)

σ_{p+}: $\tilde{\nu}_{max,abs} = -2245.4 \cdot \sigma_{p+} + 29640$ [cm^{-1}] ($R^2 = 0.8299$)

σ_{p-}: $\tilde{\nu}_{max,abs} = -2583.2 \cdot \sigma_{p-} + 30508$ [cm^{-1}] ($R^2 = 0.8081$)

σ_R: $\tilde{\nu}_{max,abs} = -4601.5 \cdot \sigma_R + 29321$ [cm^{-1}] ($R^2 = 0.5173$)

σ_{R+}: $\tilde{\nu}_{max,abs} = -2327 \cdot \sigma_{R+} + 28946$ [cm^{-1}] ($R^2 = 0.5911$)

Emission

σ_p: $\tilde{\nu}_{max,em} = -2627.5 \cdot \sigma_p + 21011$ [cm^{-1}] ($R^2 = 0.9129$)

σ_{p+}: $\tilde{\nu}_{max,em} = -1367.9 \cdot \sigma_{p+} + 21088$ [cm^{-1}] ($R^2 = 0.8272$)

σ_{p-}: $\tilde{\nu}_{max,em} = -1655.3 \cdot \sigma_{p-} + 21635$ [cm^{-1}] ($R^2 = 0.8911$)

σ_R: $\tilde{\nu}_{max,em} = -3035.6 \cdot \sigma_R + 20863$ [cm^{-1}] ($R^2 = 0.6046$)

σ_{R+}: $\tilde{\nu}_{max,em} = -1474.2 \cdot \sigma_{R+} + 20641$ [cm^{-1}] ($R^2 = 0.6371$)

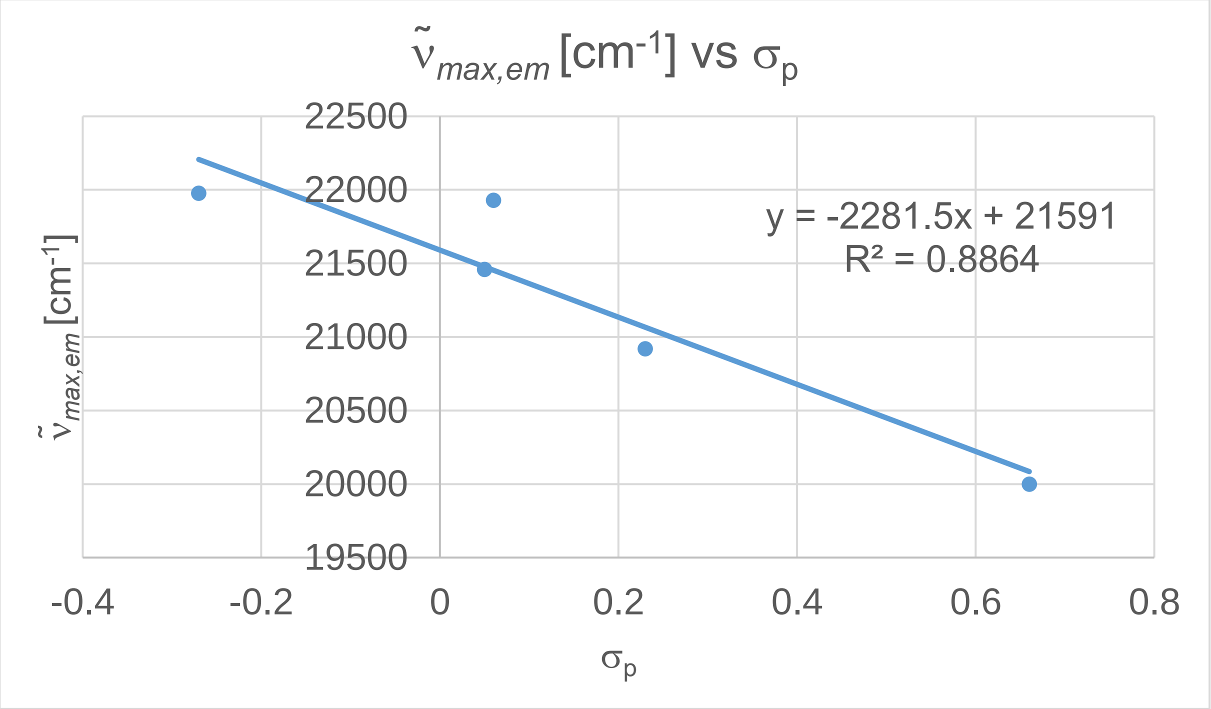

Figure S30. Hammett correlation of $\tilde{\nu}_{max,em}$ of compounds **8b-f** against σ_p.

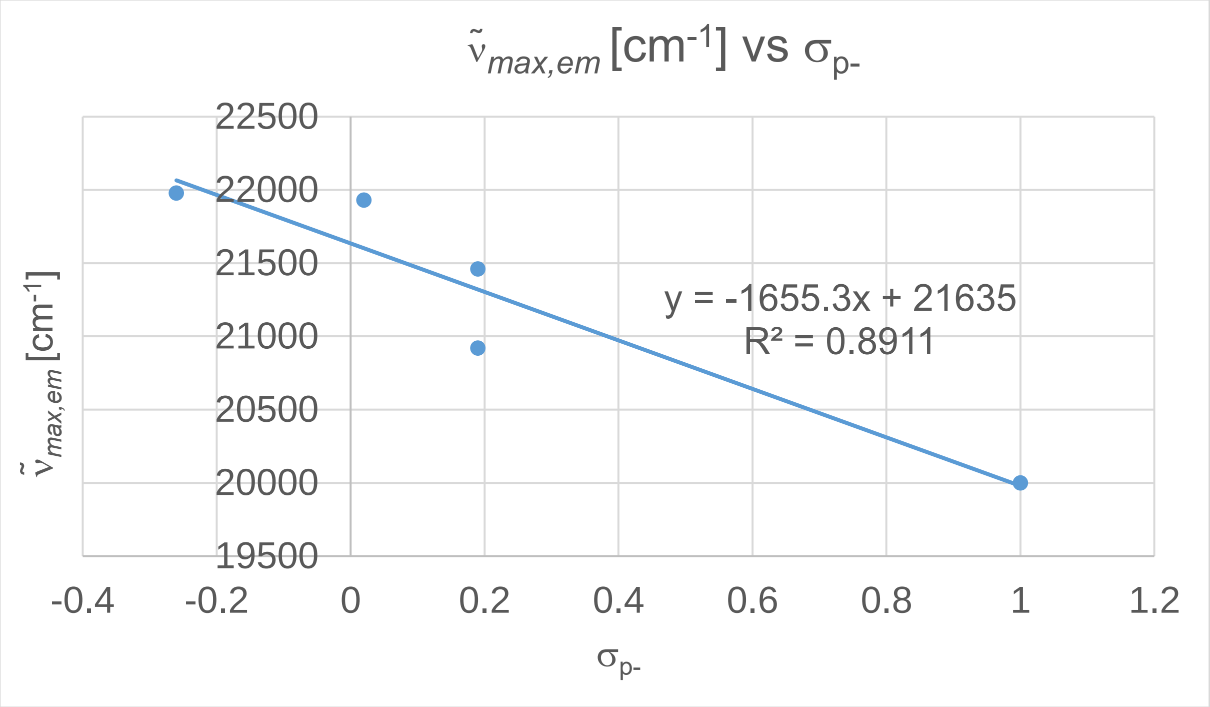

Figure S31. Hammett correlation of $\tilde{\nu}_{max,em}$ of compounds **8b-f** against σ_{p-}.

Stokes

σ_p: $\Delta\tilde{\nu} = -1348.5 \cdot \sigma_p + 8858.1$ [cm^{-1}] ($R^2 = 0.6649$)

σ_{p+}: $\Delta\tilde{\nu} = -877.41 \cdot \sigma_{p+} + 8552.4$ [cm^{-1}] ($R^2 = 0.7307$)

σ_{p-}: $\Delta\tilde{\nu} = -927.92 \cdot \sigma_{p-} + 8872.8$ [cm^{-1}] ($R^2 = 0.6013$)

σ_R: $\Delta\tilde{\nu} = -1565.9 \cdot \sigma_R + 8457.7$ [cm^{-1}] ($R^2 = 0.3454$)

σ_{R+}: $\Delta\tilde{\nu} = -852.78 \cdot \sigma_{R+} + 8304.8$ [cm^{-1}] ($R^2 = 0.4578$)
